# Global prevalence of antibiotic resistance in paediatric urinary tract infections caused by *Escherichia coli* and association with routine use of antibiotics in primary care: systematic review and meta-analysis

**DOI:** 10.1136/bmj.i939

**Published:** 2016-03-15

**Authors:** Ashley Bryce, Alastair D Hay, Isabel F Lane, Hannah V Thornton, Mandy Wootton, Céire Costelloe

**Affiliations:** 1 Centre for Academic Primary Care, NIHR School for Primary Care Research, School of Social and Community Medicine, University of Bristol, Bristol BS8 2PS, UK; 2 Specialist Antimicrobial Chemotherapy Unit, Public Health Wales Microbiology Cardiff, University Hospital of Wales, Cardiff CF14 4XW, UK; 3 NIHR Health Protection Research Unit in Healthcare Associated Infections and Antimicrobial Resistance, Imperial College London, London W12 0NN, UK

## Abstract

**Objectives** To systematically review studies investigating the prevalence of antibiotic resistance in urinary tract infections caused by *Escherichia coli* in children and, when appropriate, to meta-analyse the relation between previous antibiotics prescribed in primary care and resistance.

**Design and data analysis** Systematic review and meta-analysis. Pooled percentage prevalence of resistance to the most commonly used antibiotics in children in primary care, stratified by the OECD (Organisation for Economic Co-operation and Development) status of the study country. Random effects meta-analysis was used to quantify the association between previous exposure to antibiotics in primary care and resistance.

**Data sources** Observational and experimental studies identified through Medline, Embase, Cochrane, and ISI Web of Knowledge databases, searched for articles published up to October 2015.

**Eligibility criteria for selecting studies** Studies were eligible if they investigated and reported resistance in community acquired urinary tract infection in children and young people aged 0-17. Electronic searches with MeSH terms and text words identified 3115 papers. Two independent reviewers assessed study quality and performed data extraction.

**Results** 58 observational studies investigated 77 783 *E coli* isolates in urine. In studies from OECD countries, the pooled prevalence of resistance was 53.4% (95% confidence interval 46.0% to 60.8%) for ampicillin, 23.6% (13.9% to 32.3%) for trimethoprim, 8.2% (7.9% to 9.6%) for co-amoxiclav, and 2.1% (0.8 to 4.4%) for ciprofloxacin; nitrofurantoin was the lowest at 1.3% (0.8% to 1.7%). Resistance in studies in countries outside the OECD was significantly higher: 79.8% (73.0% to 87.7%) for ampicillin, 60.3% (40.9% to 79.0%) for co-amoxiclav, 26.8% (11.1% to 43.0%) for ciprofloxacin, and 17.0% (9.8% to 24.2%) for nitrofurantoin. There was evidence that bacterial isolates from the urinary tract from individual children who had received previous prescriptions for antibiotics in primary care were more likely to be resistant to antibiotics, and this increased risk could persist for up to six months (odds ratio 13.23, 95% confidence interval 7.84 to 22.31).

**Conclusions** Prevalence of resistance to commonly prescribed antibiotics in primary care in children with urinary tract infections caused by *E coli* is high, particularly in countries outside the OECD, where one possible explanation is the availability of antibiotics over the counter. This could render some antibiotics ineffective as first line treatments for urinary tract infection. Routine use of antibiotics in primary care contributes to antimicrobial resistance in children, which can persist for up to six months after treatment.

## Introduction

Antimicrobial resistance is an internationally recognised threat to health. The contribution of primary healthcare is particularly important as this is where almost 80% of all antibiotics used within the health service are prescribed.^1^ Bacterial infections resistant to antibiotics can limit the availability of effective treatment options, rendering some commonly encountered bacterial infections difficult to treat, including those of the urinary tract. Antibiotic resistant infections are also twice as likely to be associated with greater morbidity and mortality and are associated with increased healthcare costs.^2^ In low income countries, affordability of second line drugs and reduced access to healthcare can restrict the use of newer broad spectrum antibiotics, resulting in growing concerns for increased morbidity and mortality from antibiotic resistant infections in these countries.^3^

Children receive a lot of primary healthcare services and, as such, receive a disproportionately high number of antibiotics compared with middle aged populations.^4^ Children are also key drivers of infection within communities and can contribute to the spread of bacteria from person to person. Despite this, little research has been published describing the prevalence of bacterial resistance in children or the risk factors of importance in this group. In 2010, Costelloe and colleagues conducted a systematic review that reported strong associations between previous exposure to routinely prescribed antibiotics in primary care and antimicrobial resistance persisting for up to 12 months.^5^ Most of the contributing studies, however, were conducted in adults.

Urinary tract infections are one of the most common bacterial infections seen in primary care.^6^ In children with a suspected urinary tract infection, the most common management strategy is to treat empirically with an antibiotic while results of culture and sensitivity testing are awaited. Young children are more vulnerable to immediate and long term complications, including renal scarring and renal failure,^7^ and therefore require prompt appropriate treatment. *Escherichia coli* is responsible for over 80% of all urinary tract infections^8^ and is also the most common cause of bacteraemia and foodborne infections and a cause of meningitis in neonates.^9^

We conducted a systematic review to investigate the prevalence of resistance in community acquired *E coli* urinary tract infection to the most commonly prescribed antibiotics given to children in primary care and to quantify the relation between previous exposure to antibiotics in primary care and bacterial resistance. We stratified results by OECD (Organisation for Economic Co-operation and Development) status of the study countries as antibiotics tend to be used differently in these groups. In the more developed OECD countries antibiotics are obtained mostly only by prescription, whereas in “developing” non-OECD countries many antibiotics, including those commonly used to treat urinary tract infection, can be obtained over the counter, without the need for a prescription.^10^ ^11^ ^12^ ^13^ ^14^

## Methods

### Search strategy and selection criteria

We searched Medline, Embase, and Cochrane for articles published in any language between 1955 and October 2015. MeSH terms for these databases included “drug resistance”, “antimicrobial resistance”, “bacterial resistance”, “primary health care”, “urinary tract infections”, and “children”. MeSH terms were combined with text word searches that included “antibiotic(s)”, “primary care”, “family practice”, “ambulatory care”, “community”, “UTI”, and “urinary bacteria”. Grey and unpublished literature was searched for with ISI Web of Knowledge software and included journal articles, patents, websites, conference proceedings, government and national reports, and open access material. We screened reference lists of selected key papers and contacted authors who appeared multiple times to request details of further published and unpublished work. All full text papers were subject to citation searches. Appendix 1 details the full search strategy. Our review protocol was published on PROSPERO (www.crd.york.ac.uk/PROSPERO/).

Two independent reviewers (AB and HT) screened all titles and abstracts independently for eligibility. Studies were eligible for inclusion if they met the following criteria: investigated and reported patterns of resistance in laboratory diagnosed *E coli *positive isolates from children with urinary tract infection from primary care, defined as the first point of contact in the healthcare system; or investigated associations between previous antibiotic exposure and bacterial resistance; and study participants were children and young people aged 0-17 presenting with symptoms of urinary tract infection who had provided a urine sample. We included hospital based studies when it was clear that the investigation was for community acquired urinary tract infection, defined as a laboratory diagnosed infection from urine samples taken within 48 hours of admission.

### Data extraction and quality assessment

Full text papers for all eligible studies were obtained, and three reviewers (AB, CC, and IL) extracted data independently using a purpose built spreadsheet. The following information was extracted from each paper, when provided: author, journal, year of publication, study design, study country, economic status, participants and recruitment location, recruitment time period, age range, method of urine sample collection and testing, method of antimicrobial sensitivity testing, bacteria cultured and reported antibiotic sensitivities, previously prescribed antibiotics, and time between antibiotic exposure and urine sample collection. Level of development was measured with the OECD status of the country in which the study was conducted.^15^ The OECD is an international economic organisation first established in 1948, now made up of 34 countries, which aims to work together and with emerging and developing economies to reduce poverty through economic growth and financial stability.^15^ Member countries tend to be “developed” countries, whereas non-member countries tend to be “developing.” We used OECD status as a general measure of country level development and primary care infrastructure and a proxy marker for use of over-the-counter antibiotics. For antimicrobial exposure, time was generally recorded as a period of days, weeks, or months before the urine sample was taken and resistance was measured with standard local laboratory methods. When any information was unclear in the paper, we contacted authors for clarification.

We extracted and reported resistance to antibiotics commonly prescribed and reported for urinary tract infection in children in primary care: ampicillin, co-amoxiclav (amoxicillin-clavulanic acid), co-trimoxazole (trimethoprim-sulfamethoxazole), trimethoprim, nitrofurantoin, ciprofloxacin, and ceftazidime (as a marker for cephalosporin resistance). Ampicillin was reported in place of amoxicillin because of more frequent reporting and its equivalence in spectrum of antimicrobial activity.^16^

We used the Cochrane collaboration’s risk of bias tool to assess papers for quality.^17^ Selection bias was assessed with the Critical Appraisal Skills Programme (CASP) checklist for cohort and case-control studies (www.casp-uk.net). We produced quality assessment charts based on a traffic light system of “good,” “adequate,” and “poor” reporting (see appendix 2), as recommended by Cochrane.^17^ Our key quality criteria for eligible studies were a reliable measure of antibiotic resistance; clear reporting of bacterial resistance in children and young people aged up to 17; and clear reporting of urinary bacteria isolated as community acquired. The same key quality indicators applied for papers that included information on previous antibiotic exposure, with the addition of adjustment for confounders including age, sex, previous admission to hospital, and comorbidities.

### Data synthesis and analysis

All statistical analyses were conducted with Stata version 13 software, and all methods undertaken according to PRISMA guidelines.^18^

We calculated estimates of pooled prevalence of resistance by generating a forest plot for each antibiotic, stratified by OECD status. Forest plots illustrated the proportion of resistant *E coli* for each country, along with 95% confidence intervals, and the pooled prevalence of resistance per antibiotic per economic country group (OECD *v* non-OECD). We calculated pooled estimates for each country and for OECD and non-OECD groups using the pooled country estimates. Pooled prevalence estimates were generated for children/young people of all age groups (ages 0-17) and children aged 0-5, for comparison. When we could identify the first line antibiotics, these were indicated in the forest plot. I^2^ of 25%, 50%, and 75% were used to signify low level, moderate level, and high level heterogeneity, in line with Cochrane recommendations.^17^ All pooled estimates and 95% confidence intervals were generated with double arcsine transformation to adjust for variance instability. This avoids implausible 95% confidence intervals for prevalence estimates when generated under the normal approximation.^19^

For studies investigating the association between previous antibiotic exposure and bacterial resistance, the outcome measure was the odds ratio of bacterial resistance in children previously exposed to antibiotics compared with those children previously unexposed. The crude estimates from these studies were grouped according to the reported preceding exposure time period (0-1 month, 0-3 months, and 0-6 months). One study investigated exposure at discrete time intervals up to 12 months or more before urine sampling and was reported separately. We carried out a random effects meta-analysis and generated a pooled odds ratio for each exposure time period measured. These were compared with adjusted odds ratios for each time period, when reported. We assessed heterogeneity using the I^2^ statistic. Meta-regression was used to investigate differences in the odds ratios between antibiotic exposure and resistance across different time periods. Finally, we generated funnel plots to explore the possibility of small study effects, which can be caused by publication bias.

### Patient involvement

No patients were involved in setting the research question or the outcome measures, nor were they involved in developing plans for design or implementation of the review. No patients were asked to advise on interpretation or writing up of results. There are no plans to disseminate the results of the research to study participants or the relevant patient community.

## Results

### Study characteristics

We identified 4246 articles through database searches. Of these, we assessed 3115 non-duplicated papers and excluded 2491 on basis of title (fig 1). The 624 remaining papers were assessed by abstract screening; 540 did not meet our eligibility criteria. We obtained and assessed 84 full text papers, with 26 papers not meeting our eligibility criteria for the following reasons: 12 had no primary care data, 11 did not report antibiotic susceptibilities for *E coli* urinary tract infection bacteria, two studies were in adults, and one paper reported duplicate data from another included paper. We therefore included 58 papers in our review,^8^ ^20^ ^21^ ^22^ ^23^ ^24^ ^25^ ^26^ ^27^ ^28^ ^29^ ^30^ ^31^ ^32^ ^33^ ^34^ ^35^ ^36^ ^37^ ^38^ ^39^ ^40^ ^41^ ^42^ ^43^ ^44^ ^45^ ^46^ ^47^ ^48^ ^49^ ^50^ ^51^ ^52^ ^53^ ^54^ ^55^ ^56^ ^57^ ^58^ ^59^ ^60^ ^61^ ^62^ ^63^ ^64^ ^65^ ^66^ ^67^ ^68^ ^69^ ^70^ ^71^ ^72^ ^73^ ^74^ ^75^ ^76^ of which five papers (all from OECD countries) reported information on previous antibiotic exposure and were included in our meta-analysis. No grey literature or national reports were eligible for inclusion in the review.

**Figure f1:**
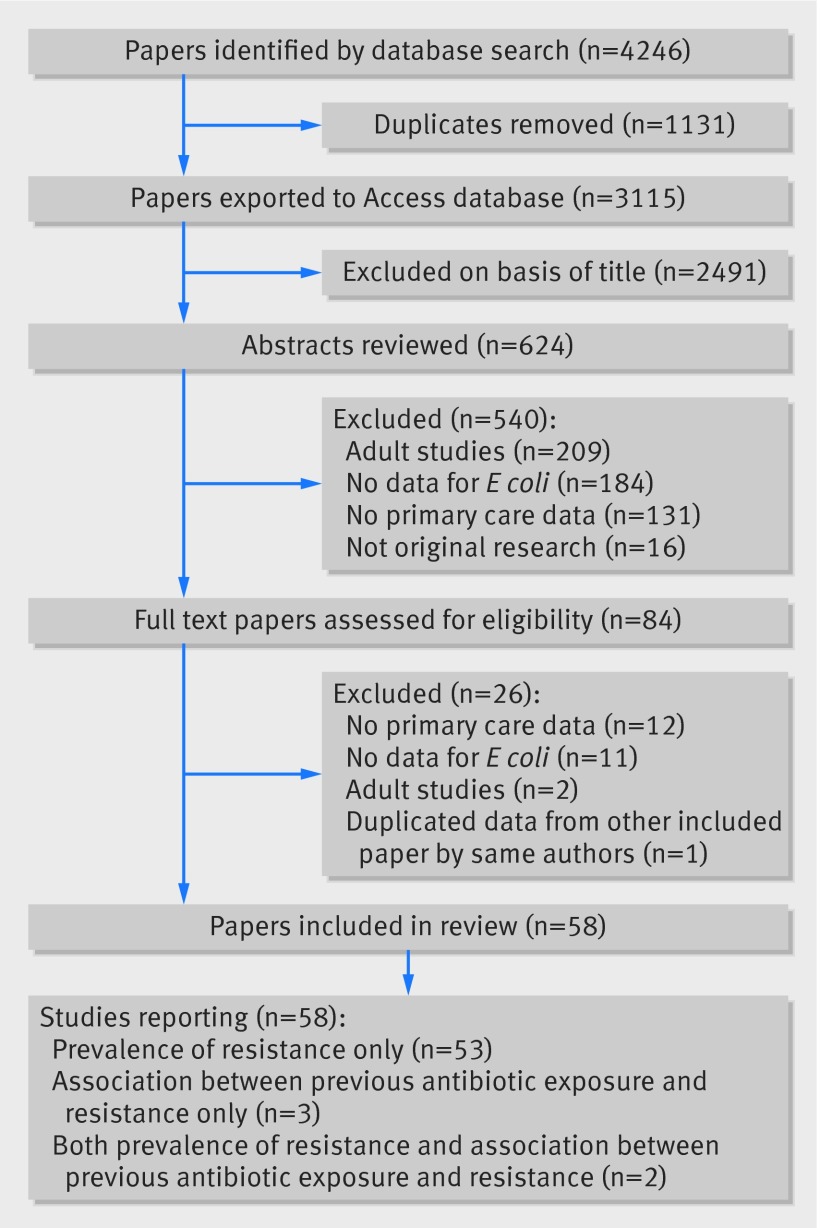
**Fig 1** Data search and extraction (PRISMA flow chart)

Table 1 summarises the characteristics of the 58 included studies (full details are in appendix 3). Thirty three studies from OECD countries reported resistance in 73 375 *E coli* isolates from the same number of children, with the exception of one UK study that included multiple urine isolates per child. As data reported in the UK study were analysed with a two level model of samples nested within patients, we reported it separately in our meta-analysis.^24^ All studies were observational; 25 were retrospective, six prospective, and two case-control. Thirty reported information on prevalence of resistance in *E coli* urinary tract infection isolates, with the three remaining reporting the association between previous antibiotic exposure and *E coli* resistance only.^22^ ^38^ ^51^Table 1 also summarises the 25 studies included from non-OECD studies that reported bacterial resistance in 4408 *E coli* isolates from the same number of children. All were observational; 10 were retrospective, 11 prospective, one case-control, and three cross sectional. All 25 non-OECD studies reported information on prevalence of resistance in urinary *E coli*. No non-OECD studies reported information on previous antibiotic exposure. Figure 2 shows the number of studies per country included in the review. Most studies were conducted in OECD countries, and there were relatively few studies from each country.

**Table 1 tbl1:** Characteristics of included papers on antibiotic resistance in paediatric *E coli* urinary tract infections by OECD (Organisation for Economic Co-operation and Development) status of study country

Study characteristics	No of papers from OECD countries (n=33)	No of papers from non-OECD countries (n=25)
Study design:		
Retrospective observational	25^8^ ^20^ ^21^ ^22^ ^23^ ^24^ ^25^ ^26^ ^27^ ^28^ ^29^ ^30^ ^31^ ^32^ ^33^ ^34^ ^35^ ^36^ ^37^ ^38^ ^39^ ^40^ ^41^ ^42^ ^43^	10^52^ ^53^ ^54^ ^55^ ^56^ ^57^ ^58^ ^59^ ^60^ ^61^
Prospective observational	6^44^ ^45^ ^46^ ^47^ ^48^ ^49^	11^62^ ^63^ ^64^ ^65^ ^66^ ^67^ ^68^ ^69^ ^70^ ^71^ ^72^
Case-control	2^50^ ^51^	1^73^
Cross-sectional	0	3^74^ ^75^ ^76^
No of children in study:		
0-100	2^35^ ^40^	7^56^ ^62^ ^64^ ^66^ ^67^ ^68^ ^76^
101-500	12^23^ ^25^ ^29^ ^32^ ^36^ ^38^ ^44^ ^45^ ^46^ ^47^ ^48^ ^49^	13^53^ ^54^ ^55^ ^58^ ^60^ ^61^ ^65^ ^69^ ^71^ ^72^ ^73^ ^74^ ^75^
501-1000	6^28^ ^30^ ^33^ ^39^ ^43^ ^50^	2^52^ ^57^
1001-10 000	7^21^ ^24^ ^27^ ^31^ ^37^ ^42^ ^51^	2^59^ ^70^
≥10 001	6^8^ ^20^ ^22^ ^26^ ^34^ ^41^	1^63^
Age range (years)*:		
0-5	9^8^ ^29^ ^31^ ^33^ ^34^ ^36^ ^43^ ^45^ ^50^	6^62^ ^65^ ^69^ ^74^ ^75^ ^76^
6-17	5^8^ ^31^ ^34^ ^36^ ^50^	0
0-17	30^8^ ^20^ ^21^ ^22^ ^23^ ^24^ ^25^ ^26^ ^27^ ^28^ ^30^ ^32^ ^33^ ^34^ ^35^ ^36^ ^37^ ^38^ ^39^ ^40^ ^41^ ^42^ ^44^ ^46^ ^47^ ^48^ ^49^ ^50^ ^51^	19^52^ ^53^ ^54^ ^55^ ^56^ ^57^ ^58^ ^59^ ^60^ ^61^ ^63^ ^64^ ^66^ ^67^ ^68^ ^70^ ^71^ ^72^ ^73^
Recruitment location:		
GP practice/paediatric office	12^21^ ^22^ ^24^ ^25^ ^26^ ^28^ ^30^ ^32^ ^34^ ^37^ ^41^ ^50^	5^53^ ^58^ ^61^ ^67^ ^70^
Outpatient/clinic	10^8^ ^27^ ^29^ ^36^ ^40^ ^42^ ^47^ ^48^ ^49^ ^51^	9^55^ ^56^ ^57^ ^59^ ^63^ ^64^ ^68^ ^71^ ^73^
Emergency department	7^20^ ^35^ ^38^ ^39^ ^43^ ^45^ ^46^	1^60^
Hospital admission	4^23^ ^31^ ^33^ ^44^	9^52^ ^54^ ^65^ ^66^ ^69^ ^72^ ^74^ ^75^ ^76^
Not reported	0	1^62^
Method of urine sampling:		
At least one of clean catch, catheter, or suprapubic aspiration	20^22^ ^23^ ^25^ ^27^ ^28^ ^29^ ^30^ ^31^ ^32^ ^33^ ^34^ ^35^ ^38^ ^39^ ^43^ ^44^ ^46^ ^47^ ^49^ ^51^	11^53^ ^54^ ^61^ ^63^ ^65^ ^67^ ^72^ ^74^ ^75^ ^76^ ^79^
Clean catch only	3^26^ ^40^ ^41^	4^59^ ^64^ ^70^ ^73^
Catheter only	1^45^	0
Suprapubic aspiration only	0	3^60^ ^62^ ^69^
Not reported	9^8^ ^20^ ^21^ ^24^ ^36^ ^37^ ^42^ ^48^ ^50^	7^52^ ^55^ ^56^ ^57^ ^66^ ^68^ ^71^
Antibiotic susceptibilities reported:		
Ampicillin	25^8^ ^20^ ^21^ ^23^ ^26^ ^28^ ^29^ ^30^ ^31^ ^32^ ^33^ ^34^ ^35^ ^36^ ^37^ ^38^ ^39^ ^40^ ^41^ ^42^ ^43^ ^44^ ^45^ ^46^ ^47^ ^48^ ^49^ ^50^	15^52^ ^53^ ^56^ ^57^ ^58^ ^61^ ^62^ ^65^ ^68^ ^69^ ^70^ ^71^ ^72^ ^73^ ^74^
Co-amoxiclav	21^8^ ^20^ ^21^ ^23^ ^25^ ^26^ ^27^ ^28^ ^29^ ^30^ ^31^ ^33^ ^34^ ^37^ ^41^ ^42^ ^44^ ^46^ ^47^ ^48^ ^49^	8^52^ ^61^ ^62^ ^69^ ^70^ ^72^ ^73^ ^74^
Co-trimoxazole	24^8^ ^20^ ^21^ ^23^ ^25^ ^27^ ^28^ ^29^ ^30^ ^31^ ^32^ ^33^ ^34^ ^36^ ^38^ ^39^ ^42^ ^44^ ^45^ ^46^ ^47^ ^48^ ^49^ ^50^	18^52^ ^53^ ^54^ ^56^ ^57^ ^58^ ^59^ ^60^ ^61^ ^64^ ^65^ ^66^ ^67^ ^69^ ^72^ ^73^ ^74^ ^76^
Trimethoprim	7^24^ ^26^ ^33^ ^35^ ^37^ ^41^ ^43^	1^70^
Nitrofurantoin	21^8^ ^20^ ^25^ ^26^ ^27^ ^28^ ^32^ ^33^ ^35^ ^37^ ^38^ ^39^ ^41^ ^42^ ^43^ ^44^ ^45^ ^46^ ^47^ ^49^ ^50^	18^53^ ^54^ ^57^ ^58^ ^59^ ^60^ ^61^ ^62^ ^64^ ^66^ ^67^ ^68^ ^69^ ^70^ ^72^ ^73^ ^75^ ^76^
Ciprofloxacin	17^8^ ^20^ ^25^ ^26^ ^27^ ^28^ ^29^ ^30^ ^31^ ^32^ ^33^ ^35^ ^37^ ^41^ ^42^ ^45^ ^46^ ^47^ ^48^	11^52^ ^55^ ^56^ ^58^ ^59^ ^61^ ^62^ ^63^ ^64^ ^66^ ^68^
Ceftazidime	10^20^ ^25^ ^28^ ^29^ ^31^ ^39^ ^41^ ^45^ ^46^ ^48^	8^52^ ^53^ ^55^ ^56^ ^62^ ^70^ ^73^ ^75^
Method of antimicrobial susceptibility testing:		
Disk diffusion	23^20^ ^21^ ^23^ ^25^ ^27^ ^29^ ^31^ ^32^ ^33^ ^35^ ^36^ ^37^ ^39^ ^40^ ^41^ ^42^ ^43^ ^44^ ^45^ ^47^ ^48^ ^49^ ^51^	21^52^ ^53^ ^54^ ^55^ ^56^ ^57^ ^58^ ^59^ ^60^ ^63^ ^65^ ^66^ ^67^ ^68^ ^69^ ^70^ ^71^ ^72^ ^73^ ^74^ ^75^ ^76^
Minimum inhibitory concentration	2^8^ ^34^	0
Vitek	3^26^ ^28^ ^50^	0
Not reported	5^22^ ^24^ ^30^ ^38^ ^46^	4^55^ ^61^ ^62^ ^64^
Guidelines used to interpret antimicrobial sensitivities:		
CLSI	25^8^ ^20^ ^21^ ^22^ ^23^ ^25^ ^26^ ^27^ ^28^ ^29^ ^31^ ^32^ ^34^ ^35^ ^36^ ^38^ ^39^ ^41^ ^43^ ^44^ ^47^ ^48^ ^49^ ^50^ ^51^	18^53^ ^54^ ^56^ ^57^ ^58^ ^60^ ^63^ ^64^ ^65^ ^66^ ^67^ ^68^ ^69^ ^71^ ^72^ ^74^ ^75^ ^76^
BSAC	1^37^	0
Not reported	7^24^ ^30^ ^33^ ^40^ ^42^ ^45^ ^46^	7^52^ ^55^ ^59^ ^61^ ^62^ ^70^ ^73^
Previous antibiotic exposure information†	5^22^ ^24^ ^38^ ^50^ ^51^	0

CLSI=Clinical Laboratory Standards Institute; BSAC=British Standard for Antimicrobial Chemotherapy.

*Age 0-5: papers that report data specifically for this age group; 6-17: papers that report data specifically for this age group; 0-17: papers that which report data for children/young people within 0-17 and do not fit into previous reported age groups. Papers can appear more than once depending on how results are reported.

†No studies from non-OECD countries collected previous antibiotic exposure data and were not included in meta-analysis.

**Figure f2:**
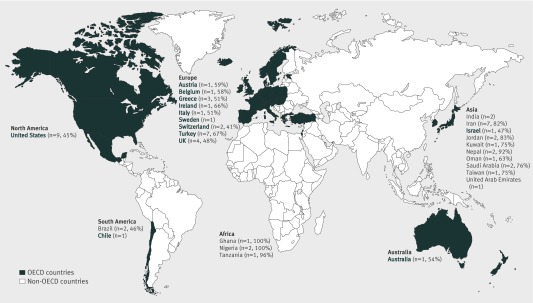
**Fig 2** Geographical distribution of urinary *E coli* resistance prevalence to ampicillin (%) by OECD and non-OECD countries,^15^ with number of included studies per country in parentheses)

Thirty one (20 OECD *v* 11 non-OECD) studies used mixed methods for urine collection, including clean catch, catheter, or suprapubic aspiration, with the remaining studies using a single method. There were no differences in rates of resistance detected between the different methods of urine sampling that studies used. Antimicrobial sensitivity testing was carried out with standard disk diffusion methods in 44 studies, with one interpreted and reported according to British Standard for Antimicrobial Chemotherapy (BSAC), 43 with Clinical and Laboratory Standards Institute (CLSI) guidelines,^77^ ^78^ and 14 not reported. There were no differences in resistance between studies that did and did not report the use of antimicrobial sensitivity guidelines. All children had presented to primary care facilities (18 studies), outpatient clinics (19 studies), or emergency departments (eight studies) with symptoms of a urinary tract infection, with some children sent to a secondary or tertiary care hospital for urine tests on admission (12 studies). Of the 12 inpatient studies, nine stated that they included only community acquired *E coli* isolates; the three remaining confirmed urine samples were collected within 48 hours of admission.

The quality assessment “traffic light” charts for the included studies showed that, for the five studies reporting information on antibiotic exposure, reporting was generally good for our all our key quality indicators (appendix 2). For studies reporting prevalence of resistance only, overall quality was good with the exception of adjustment for confounding.

### Prevalence of resistance in urinary *E coli*

Table 2 shows the prevalence of *E coli* urinary isolates resistance to antibiotics. These data were obtained from forest plots generated for each antibiotic (appendix 4).

**Table 2 tbl2:** Pooled percentage prevalence (95% confidence interval) of resistance to antibiotics in primary care used to treat urinary *E coli* infection in children (see appendix 4 for corresponding forest plots) by OECD (Organisation for Economic Co-operation and Development) status of study country

Antibiotic	OECD					Non-OECD			
	Pooled prevalence (%)	No of isolates tested	No of reporting studies	I^2^ (%)		Pooled prevalence (%)	No of isolates tested	No of reporting studies	I^2^ (%)
Ampicillin	53.4 (46.0 to 60.8)	66 503	25 (11 countries)^8^ ^20^ ^21^ ^23^ ^26^ ^28^ ^29^ ^30^ ^31^ ^32^ ^33^ ^34^ ^35^ ^37^ ^38^ ^39^ ^40^ ^41^ ^42^ ^44^ ^46^ ^47^ ^48^ ^49^ ^50^	7		79.8 (73.0 to 87.7)	2265	15 (11 countries)^52^ ^53^ ^56^ ^57^ ^58^ ^61^ ^62^ ^65^ ^68^ ^69^ ^70^ ^71^ ^72^ ^73^ ^74^	25
Co-amoxiclav	8.2 (7.9 to 9.6)	65 076	21 (9 countries)^8^ ^20^ ^21^ ^23^ ^25^ ^26^ ^27^ ^28^ ^29^ ^30^ ^31^ ^33^ ^34^ ^37^ ^41^ ^42^ ^44^ ^46^ ^47^ ^48^ ^49^	45		60.3 (40.9 to 79.0)	1256	8 (8 countries)^52^ ^61^ ^62^ ^69^ ^70^ ^72^ ^73^ ^74^	62
Co-trimoxazole	30.2 (20.5 to 39.3)	50 230	24 (9 countries)^8^ ^20^ ^21^ ^23^ ^25^ ^27^ ^28^ ^29^ ^30^ ^31^ ^32^ ^33^ ^34^ ^36^ ^38^ ^39^ ^42^ ^44^ ^45^ ^46^ ^47^ ^48^ ^49^ ^50^	28		69.6 (59.8 to 81.5)	2590	18 (10 countries)^52^ ^53^ ^54^ ^56^ ^57^ ^58^ ^59^ ^60^ ^61^ ^64^ ^65^ ^66^ ^67^ ^69^ ^72^ ^73^ ^74^ ^76^	37
Trimethoprim	23.6 (13.9 to 32.3)	18 977	7 (5 countries)^24^ ^26^ ^33^ ^35^ ^37^ ^41^ ^43^	16		Too few data*	596	1 (1 country)^70^	Too few data*
Nitrofurantoin	1.3 (0.8 to 1.7)	50 994	21 (13 countries)^8^ ^20^ ^25^ ^26^ ^27^ ^28^ ^32^ ^33^ ^35^ ^37^ ^38^ ^39^ ^40^ ^41^ ^42^ ^43^ ^44^ ^45^ ^46^ ^47^ ^49^ ^50^	0		17.0 (9.8 to 24.2)	3020	18 (10 countries)^53^ ^54^ ^57^ ^58^ ^59^ ^60^ ^61^ ^62^ ^64^ ^66^ ^67^ ^68^ ^69^ ^70^ ^72^ ^73^ ^75^ ^76^	42
Ciprofloxacin	2.1 (0.8 to 4.4)	52 209	17 (9 countries)^8^ ^20^ ^25^ ^26^ ^27^ ^28^ ^31^ ^32^ ^33^ ^35^ ^37^ ^41^ ^42^ ^45^ ^46^ ^47^ ^48^	59		26.8 (11.1 to 43.0)	1723	11 (7 countries)^52^ ^55^ ^56^ ^58^ ^59^ ^61^ ^62^ ^63^ ^64^ ^66^ ^68^	35
Ceftazidime†	2.4 (0.9 to 3.3)	25 805	10 (8 countries)^20^ ^25^ ^28^ ^29^ ^31^ ^39^ ^41^ ^45^ ^46^ ^48^	58		26.1 (14.6 to 37.5)	1136	8 (5 countries)^52^ ^53^ ^55^ ^56^ ^62^ ^70^ ^73^ ^75^	54

*Only one study from non-OECD countries (Saudi-Arabia).

†Marker for cephalosporin resistance.

For all antibiotics tested, the prevalence of antibiotic resistance was higher in non-OECD than in OECD countries. For all countries the prevalence of resistance was highest for ampicillin. Figure 2 shows the pooled prevalence (or single study reported prevalence if n=1) of ampicillin resistance by country. Switzerland had the lowest prevalence at 41%, with Ghana and Nigeria highest at 100%.

Pooled prevalences of resistance to co-trimoxazole and trimethoprim were high in OECD countries, with co-trimoxazole resistance at 30%. Resistance to co-trimoxazole was more than twice as high in non-OECD compared with OECD countries. Trimethoprim resistance was reported in only one non-OECD study, conducted by Al-Mugeiren and colleagues in Saudi-Arabia, which reported 67% resistance from 596 isolates.^70^ Nitrofurantoin resistance was the lowest of all reported for all countries.

Pooled prevalences of resistance to ciprofloxacin and ceftazidime in children’s *E coli* urinary isolates were both around 2% in OECD countries; however, resistance to both antibiotics was over 10 times higher in non-OECD countries, both over 26% (table 2).

When we stratified by “reported first line” antibiotic versus “first line not specified” for each country, estimates of prevalence of resistance for OECD countries were similar to overall OECD estimates reported in table 2, with little difference in estimates when first line treatment was specified or not. In non-OECD countries, however, pooled estimates of resistance were generally higher for those countries that specified the antibiotic as first line. The difference was particularly large for co-trimoxazole (first line pooled resistance 76.2% (95% confidence interval 64.1% to 87.2%) versus non-first line resistance 55.6% (26.6% to 84.7%)) and ciprofloxacin (first line pooled resistance 58.1% (51.5% to 64.7%) versus non-first-line pooled resistance 15.8% (4.7% to 26.8%) (appendix 5).

### Prevalence of resistance in children aged 0-5

Twelve studies reported resistance in urinary *E coli* specifically for children aged 0-5, seven from OECD countries and five from non-OECD countries (table 3). As there were insufficient data to compare with children and young people aged 6-17, we compared these data with data from all children (table 2). As with all children, the prevalence of antibiotic resistance in children aged 0-5 was higher in non-OECD than OECD countries. Compared with the data for all children, in OECD countries the pooled prevalence of resistance in urinary *E coli* in children aged 0-5 was higher for ampicillin and ceftazidime, and lower for co-amoxiclav, co-trimoxazole, and nitrofurantoin (table 2). In non-OECD countries, resistance was higher against all reported antibiotics for children aged 0-5 compared with all children.

**Table 3 tbl3:** Pooled prevalence (%) of resistance to antibiotics in primary care used to treat urinary *E coli* infection in children aged 0-5 by OECD (Organisation for Economic Co-operation and Development) status of study country

Antibiotic	OECD					Non-OECD			
	Pooled prevalence (%)	No of isolates tested	No of reporting studies	I^2^ (%)		Pooled prevalence (%)	No of isolates tested	No of reporting studies	I^2^ (%)
Ampicillin	55.0 (48.6 to 61.4)	5273	5 (4 countries)^8^ ^29^ ^31^ ^33^ ^34^	10		90.3 (73.4 to 100)	176	3 (3 countries)^65^ ^69^ ^74^	0
Co-amoxiclav	9.6 (5.7 to 13.5)	5273	5 (4 countries)^8^ ^29^ ^31^ ^33^ ^34^	51		71.9 (40.7 to 100)	89	3 (3 countries)^62^ ^69^ ^74^	66
Co-trimoxazole	29.8 (21.0 to 38.5)	5405	7 (5 countries)^8^ ^29^ ^31^ ^33^ ^34^ ^36^ ^45^	39		71.0 (44.9 to 97.0)	257	5 (4 countries)^65^ ^69^ ^74^ ^75^ ^76^	0
Trimethoprim	Too few data*	188	1 (1 country)^33^	Too few data*		No data†	0	0	—
Nitrofurantoin	0.4 (0.0 to 0.7)	3089	5 (5 countries)^8^ ^33^ ^29^ ^43^ ^45^	45		35.2 (31.6 to 38.8)	145	3 (3 countries)^62^ ^69^ ^75^	0
Ciprofloxacin	6.2 (3.2 to 9.3)	4544	4 (4 countries)^8^ ^31^ ^33^ ^45^	33		Too few data‡	49	1 (1 country)^62^	Too few data^c^
Ceftazidime§	4.9 (0.3 to 9.5)	1535	4 (4 countries)^29^ ^31^ ^33^ ^45^	28		43.6 (9.0 to 78.2)	130	2 (2 countries)^62^ ^75^	0

*Only one study from OECD countries (Austria).

†No studies from non-OECD countries reported resistance to trimethoprim in children aged 0-5.

‡Only one study from non-OECD countries (India).

§Marker for cephalosporin resistance.

### Association between previous antibiotic exposure and bacterial resistance

Figure 3 shows a forest plot of five studies investigating the relation between previous exposure to any versus no antibiotics and bacterial resistance. The studies varied in the combinations of antibiotic resistance and exposure investigated, some reporting resistance and exposure to any antibiotic, while others reported resistance to trimethoprim, co-trimoxazole, or third generation cephalosporins. In figure 3, for all time periods of exposure the crude odds of resistance were greater in children exposed to antibiotics than in those who were unexposed. The effect sizes show a decline between exposure time periods of 0-1 month (pooled odds ratio 8.38, 95% confidence interval 2.84 to 24.77) and 0-3 months (3.38, 2.05 to 5.55), then an increase at 0-6 months (13.23, 7.84 to 22.31). The study by Allen and colleagues, which explored the association between exposure to any antibiotic in the previous six months and resistance to co-trimoxazole, measured previous exposure to antibiotics for four weeks or more within the six months before the urine sample was taken. The three other studies shown in figure 3 measured exposure to any antibiotic for an unspecified total prescription time period. Given the overlap in the exposure time periods reported by the included studies, we did not conduct a meta-regression analysis for the data presented in figure 3.

**Figure f3:**
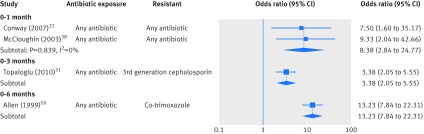
**Fig 3** Pooled crude odds ratios (log scale) for resistance in children’s urinary bacteria and previous exposure to any antibiotic. Studies grouped according to time period after antibiotic use during which exposure was measured and ordered within each time period by increasing standard error

There was no evidence of heterogeneity within groups in the 0-1 month time period, with too few studies in the 0-3 and 0-6 months time periods to calculate heterogeneity. Of the four studies included in figure 3, three reported odds ratios adjusted for sex, age, race, renal abnormalities, and previous admission to hospital. The adjusted odds ratio did not differ substantially from the crude pooled estimates.

The study by Duffy and colleagues was the only one of those measuring the association between antibiotic exposure and resistance to report results based on multiple urinary isolates per child and with a more accurate measure of antibiotic exposure^24^; therefore we chose to report this study separately. Figure 4 shows the crude multilevel odds ratios for resistance to trimethoprim relative to the number of days since exposure to trimethoprim. Duffy and colleagues reported multilevel crude odds ratios for the association between exposure and resistance to trimethoprim, based on the number of urinary isolates reported in the paper not individual patients, along with the number of isolates with reported exposure to trimethoprim only for each time period. The sample level variables included in the model were age at test, time since most recent trimethoprim prescription, and year of test; patient level variables included sex, socioeconomic status, rurality, and total number of *E coli* isolates in the study period. The crude odds ratios in figure 4 show a decrease in resistance to trimethoprim with increasing time since exposure to trimethoprim. We conducted a meta-regression analysis on the crude odds ratios calculated from the paper by Duffy and colleagues, which showed a β coefficient of −0.4 (95% confidence interval −0.61 to −0.19), indicating an important time trend.

**Figure f4:**
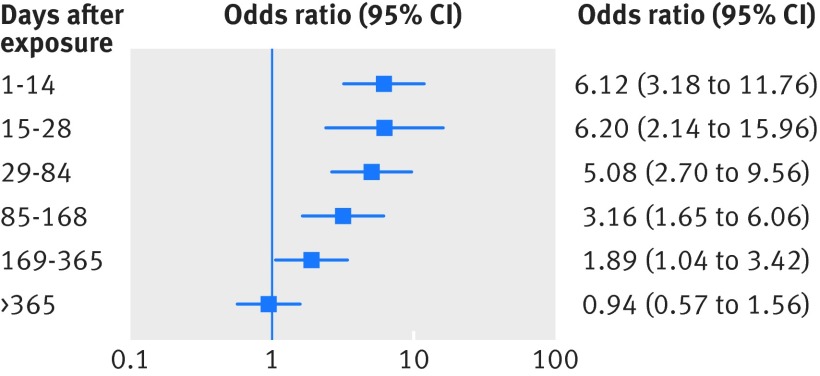
**Fig 4** Individual crude multilevel odds ratios for trimethoprim resistance in urinary isolates of children from Duffy and colleagues^24^ and previous trimethoprim prescribing

### Publication bias

There were too few studies to assess publication bias.

## Discussion

### Principal findings

The 58 studies from both OECD and non-OECD countries provide evidence of high rates of bacterial resistance in *E coli* isolates from children with urinary tract infection to some of the most commonly prescribed antibiotics in primary care. Worldwide, rates of resistance to ampicillin were the highest and nitrofurantoin rates lowest, irrespective of OECD status. Resistance to all reported antibiotics was higher in non-OECD than OECD countries, with resistance rates higher to first line than to non-first line antibiotics. Resistance could render several first line antibiotics ineffective in some countries. Prescribing of antibiotics to individual children in primary care is an important contributor to bacterial resistance, which can persist for up to six months after prescription.

### Strengths and limitations

To our knowledge, this is the first systematic review and meta-analysis to explore and report global evidence regarding the prevalence of bacterial resistance in children’s urinary tract infection and associations with the routine use of antibiotics in primary care. WHO recently published their “global action plan” on antimicrobial resistance, which described data relating to the prevalence of resistance, including geographical patterns, as a key gap in our current knowledge,^80^ which this systematic review in part fills. Our review was rigorously conducted according to the Cochrane guidelines.^17^ We chose to stratify our results by OECD status to reflect both national development and likely availability of over-the-counter antibiotics.^3^ ^81^

We are, however, aware of several limitations. Firstly, antibiotics are used differently within OECD and non-OECD countries,^82^ ^83^ ^84^ ^85^ and over-the-counter use is difficult to measure. A 2011 systematic review reported high worldwide variability in non-prescription antibiotics,^81^ with some evidence of less than 100% agreement between OECD status and over-the-counter availability. To our knowledge there is no better country level alternative, and none of the included studies reported or measured availability of over-the-counter antibiotics. We also acknowledge that factors other than antibiotic use and over-the-counter availability can account for differences in resistance prevalence between OECD and non-OECD countries, including poor sanitation, unstable governance, and lower levels of regulation of medicines. Although it is useful to explore changes in resistance over time, (for example, to understand the impact of vaccination), we were also unable to explore this as the data collected overlapped in terms of recruitment periods.

Of the five studies we included in our meta-analysis, most reported the association between previous antibiotic exposure and resistance within overlapping time periods. This implies that the associations with longer time periods (such as 0-6 months) could reflect a combination of long and short term associations. The odds ratios were highest in the 0-6 months time period; probably because this individual study measured exposure to antibiotics for a combined total of four weeks or more within the previous six months compared with no exposure within the six month time period.^68^ The other studies measured exposure to antibiotics within an undefined combined length of prescription time within the measured time period versus no antibiotic prescriptions. None of the studies we included in our meta-analysis reported antibiotic doses, so we were unable to evaluate any dose-response effects.

In most countries it is standard practice to treat empirically with an antibiotic when a child presents to primary care with a suspected urinary tract infection. Sometimes a urine sample is taken only if the illness does not respond to first line antibiotic treatment. This can lead to falsely high reported resistance rates to first line antibiotics. This problem would be removed if only incident cases were included or systematic urine sampling was used, but studies did not present this information. That said, there were no obvious differences in resistance rates according to the timing of the urine sampling. Furthermore, variation in sampling strategies could explain some of the variation in pooled prevalence of resistance between OECD and non-OECD countries, though this could not be explored from the data available. Reverse causality and other confounding associations could also have introduced bias to our findings; including previous hospital admissions, comorbidities, age, and sex. Studies that attempted to adjust for confounding factors, however, did not show differences between crude and adjusted estimates of association.

### Results in the context of existing research

#### Prevalence of urinary bacterial resistance

We believe our rates of prevalence of resistance are accurate as they are consistent with other data sources. The highest reported resistance to ampicillin in our review was similar to the reported aminopenicillin group resistance in the European EARS-Net database and US Centre for Disease Dynamics, Economics and Policy (CDDEP) databases.^86^ ^87^ Resistance to ampicillin in other studies from the US ranged between 36% and 54%, suggesting that resistance to antibiotics in young children is similar to that of the general population. The similarities observed here could be a result of transmission between age groups of genetic resistance factors such as plasmids, facilitated through frequent interaction between children and adults. Trimethoprim resistance was reported by three studies from the UK, all with large sample sizes (>1700 isolates); all reported resistance in excess of 20%. These are similar to levels of trimethoprim resistance reported by other UK based studies; Bean and colleagues reported trimethoprim resistance in community acquired urinary isolates from adults and children at 39%.^88^ Additionally, Farrell and colleagues reported 27% resistance in *E coli* urinary isolates from all age groups.^89^ In total, seven OECD studies from five countries (UK, Ireland, Austria, Australia, and Sweden) reported susceptibility to trimethoprim; these were also the only countries to report trimethoprim as a first line antibiotic treatment for urinary tract infection (appendix 5). Trimethoprim resistance was infrequently tested for in many studies from OECD countries, which probably because it is not a first line treatment in their country. Co-trimoxazole was the most common first line treatment for urinary tract infection worldwide (15 countries and 37 studies). Resistance to co-trimoxazole was relatively high worldwide, particularly in non-OECD countries at 64%. Resistance to nitrofurantoin, an antibiotic used almost exclusively for urinary tract infections, was low worldwide, supporting its continued effectiveness as a first line treatment for uncomplicated infections.^90^ ^91^ ^92^

For many of the antibiotics reported in this review, the pooled prevalence of resistance was higher in children aged 0-5 than in all children and young people (0-17). It has been previously suggested that resistance levels are likely to be higher in those communities with a higher proportion of young children because of their high consumption of antibiotics.^93^ A study conducted in France found that children aged under 7 consumed three times more antibiotics than older populations.^94^ The findings in our review support this theory as resistance to all commonly prescribed antibiotics worldwide was higher in younger children than in children of predominantly older age. Our findings also suggest there could be a reversible element of antibiotic resistance, whereby reduced use of antibiotics (in older children) reduces the selective pressure that favours antibiotic resistant strains.

#### Association between previous antibiotic exposure and bacterial resistance

Our meta-analysis showing an association between exposure to antibiotics in the previous six months and isolation of resistant urinary isolates is similar to our previous 2010 review, which explored the effect of antibiotic prescribing in primary care on the development of resistance in individual patients of all ages.^5^ Consistent with our previous review, we found some evidence from Duffy and colleagues of decreasing resistance for increasing time from antibiotic prescribing.^24^

### Policy, clinical, and research implications

Our findings detail global high level resistance to some of the most commonly prescribed antibiotics for children primary care, which could result in several drugs becoming ineffective first line treatments in many countries. The Infectious Diseases Society of America (IDSA) in collaboration with the European Society for Microbiology and Infectious Diseases (ESCMID)^95^ recommend that an antibiotic should be selected for first line empirical treatment of urinary tract infection only if the local prevalence of resistance is less than 20%. According to these guidelines, our review suggests ampicillin, co-trimoxazole, and trimethoprim are no longer suitable first line treatment options for urinary tract infection in many OECD countries and that as a result many guidelines, such as those published by the National Institute for Health and Care Excellence (NICE), might need updating. In non-OECD countries, resistance to all first line antibiotics specified for urinary tract infections was in excess of 20% (appendix 5), suggesting that choices of first line treatment might need to be re-evaluated in less well developed countries. Our results also support the need for prescribing guidelines to reflect patterns of local resistance and that, for many areas, nitrofurantoin might be the most appropriate first line treatment for lower urinary tract infection. That said, care is needed because ruling out the use of some first line antibiotics could lead clinicians to prescribe broad spectrum second line antibiotics, such as co-amoxiclav, cephalosporins, and quinolones, resulting in a vicious cycle of increasing use of broad spectrum antibiotics and bacterial resistance.

Prevalence of resistance to common antibiotics in primary care was higher in non-OECD countries than OECD countries, which could be because of weaker infrastructure of primary care, weaker regulation of antibiotic use, and the need for higher use of antibiotic because of higher risks of serious bacterial infection in children living in non-OECD countries. Improved infrastructure of primary care, access to healthcare, and antibiotic regulation might be necessary to reduce the burden of antimicrobial resistance in these settings.

Furthermore, the results indicate that bacterial resistance to antibiotics can persist for up to six months after antibiotic exposure in individual children. The study conducted by Duffy and colleagues is an exemplar of how future studies should measure associations between resistance and time since exposure to antibiotics.^24^ In addition, future studies should also consider inclusion of incident data whenever possible to facilitate better comparison with other studies. Primary care clinicians should consider the impact of any antibiotic use on subsequent antimicrobial resistance and avoid their unnecessary use by following local and national guidance whenever possible. When antibiotic treatment is needed, our findings suggest that clinicians should consider a child’s antibiotic use in the past six months when selecting further treatment, avoiding the use of broad spectrum antibiotics whenever possible.^96^ Our findings also support other evidence for the continued availability of nitrofurantoin as an effective treatment for uncomplicated urinary tract infections in primary care.^91^ ^97^

### Conclusions

Prevalence of resistance to commonly prescribed primary care antibiotics in *E coli* urinary tract infections in children is high, particularly in non-OECD countries, where one possible explanation is availability of antibiotics over the counter. This could render some drugs ineffective as first line treatments for urinary tract infection. Routine use of antibiotics in primary care contributes to antimicrobial resistance in children, which can persist for up to six months after antibiotic prescription.

**What is already known on this topic**

Throughout the world, children are prescribed a lot of antibiotics in primary care
Such routine use increases the probability of antibiotic resistance in adults with urinary tract infections
Substantial variations in antibiotic use exist globally, with over-the-counter availability common in many countries

**What this study adds**

Prevalence of antibiotic resistance in urinary tract infection in children caused by *E coli* is high globally, including to some first line treatments such as trimethoprim
Several antibiotics for children commonly used in primary care, including ampicillin (amoxicillin) and trimethoprim, could be ineffective first line treatment options
Urinary tract bacterial isolates from individual children with previous primary care prescriptions for antibiotic were more likely to be resistant to treatment, and this increased risk can persist for up to six months

## Web Extra

Extra material supplied by the author

Appendix 1: Medline and Embase search strategy

Appendix 2: Data quality charts (by studies reporting prevalence of resistance only and prevalence plus antibiotic exposure)

Appendix 3: Study characteristics

Appendix 4: Supplementary forest plots

Appendix 5: Prevalence of resistance by country level reported first line antibiotic treatment for urinary tract infection in OECD and non-OECD countries

## Notes

CC is affiliated with the National Institute for Health Research Health Protection Research Unit (NIHR HPRU) in Healthcare Associated Infection and Antimicrobial Resistance at Imperial College London in partnership with Public Health England (PHE). The views expressed are those of the author(s) and not necessarily those of the NHS, the NIHR, the Department of Health, or Public Health England.

Contributors: ADH and CC conceived and secured funding for the study. AB performed the searches. AB and HVT identified eligible studies. AB, CC, HVT, and ADH appraised study quality; data were extracted by AB, CC, and IFL. AB and CC transformed data and performed the meta-analyses. MW made substantial contributions to the overall study design and the presentation of results. AB, CC, and ADH drafted first sections of the text. All authors contributed to, reviewed, and approved the final draft. All authors received access to all the data (including statistical reports and tables) in the study and take responsibility for the integrity of the data and the accuracy of the data analysis. AB is guarantor.

Ethical approval: Not required.

Transparency: AB affirms that the manuscript is an honest, accurate, and transparent account of the study being reported; that no important aspects of the study have been omitted, and that any discrepancies from the study as planned have been explained.

Data sharing: No additional data available.
