# Supplementary material for: Global prevalence of antibiotic resistance in paediatric urinary tract infections caused by Escherichia coli and association with routine use of antibiotics in primary care: systematic review and meta-analysis
Source: BMJ. 2016 Mar 15;352:i939. doi: 10.1136/bmj.i939 (PMC4793155; doi:10.1136/bmj.i939)
Supplement: Supplementary file 1 — Appendix 1: Medline and Embase search strategy [file brya027820.ww1_default.pdf]

Appendix 1. Medline and Embase search strategy [posted as supplied by author]

MEDLINE and EMBASE search strategy	
1. exp Drug Resistance, Microbial	21. Exp. Urinary Tract Infections/ Transmission
2. Anti-bacterial Agents/ Therapeutic use	22. Exp. Urinary Tract Infections/ Microbiology
3. Antibiotic$.tw	23. Escherichia coli Infections/ Epidemiology
4. Antimicrobial$.tw	24. urinary tract infection.mp
5. antimicrobial resistance.mp	25. UTI.tw
6. resistan$.tw	26. urinary isolate$.tw
7. 1 or 2 or 3 or 4 or 5 or 6	27. uropathoge$.tw
8. Exp. Primary Health Care	28. urine.tw
9. Exp. Community-acquired Infections/ Microbiology	29. urinary.tw
10. Exp. Community-acquired Infections/ Transmission	30. 18 or 19 or 20 or 21 or 22 or 23 or 24 or 25 or 26 or 27 or 28 or 29
11. Exp. Community-acquired Infections/ Epidemiology	31. Exp. Child
12. Outpatient$.tw	32. Exp. Child/ Preschool
13. Community.tw	33. Exp. Infant
14. Family practice.mp	34. Exp. Adolescent
15. Ambulatory care.mp	35. child.tw
16. Primary care.mp	36. children.tw
17. 8 or 9 or 10 or 11 or 12 or 13 or 14 of 15 or 16	37. p?ediatri$.tw
18. Exp. Urinary Tract Infections/ Diagnosis	38. 31 or 32 or 33 or 34 or 35 or 36 or 37
19. Exp. Urinary Tract Infections/ Epidemiology	39. 7 and 17 and 30 and 38
20. Exp. Urinary Tract Infections/ Prevention and Control	
