# Supplementary material for: Global prevalence of antibiotic resistance in paediatric urinary tract infections caused by Escherichia coli and association with routine use of antibiotics in primary care: systematic review and meta-analysis
Source: BMJ. 2016 Mar 15;352:i939. doi: 10.1136/bmj.i939 (PMC4793155; doi:10.1136/bmj.i939)

Appendix 2. Data quality charts (split by studies reporting prevalence of resistance only and prevalence plus antibiotic exposure) [posted as supplied by author]

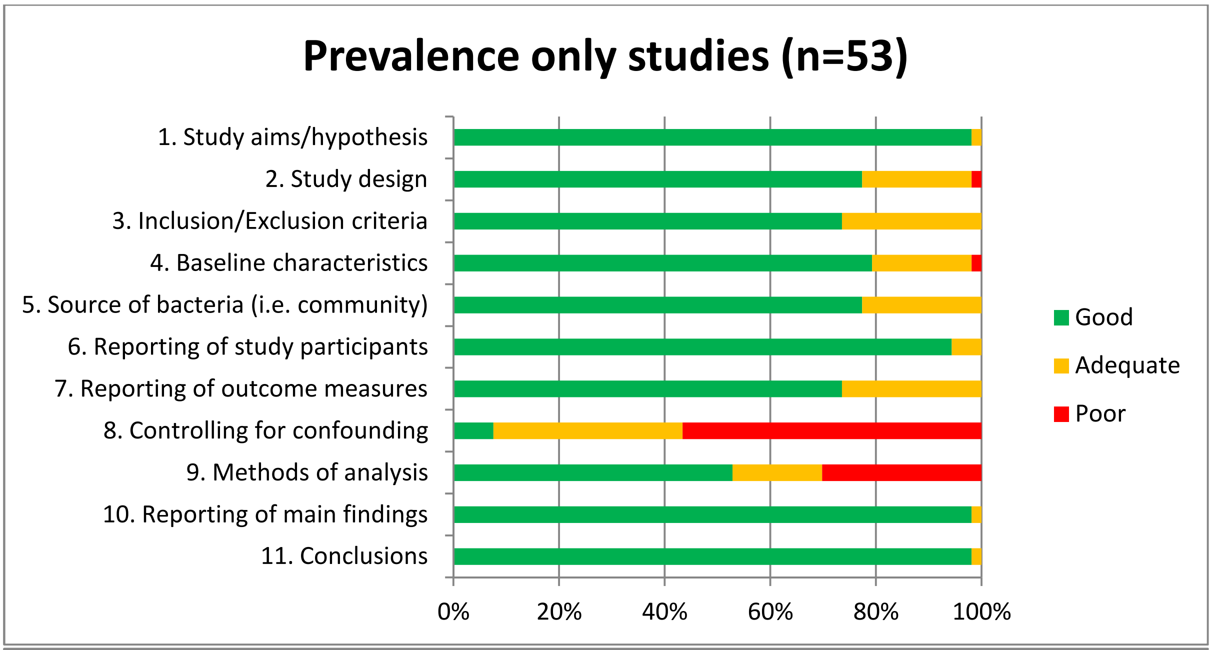

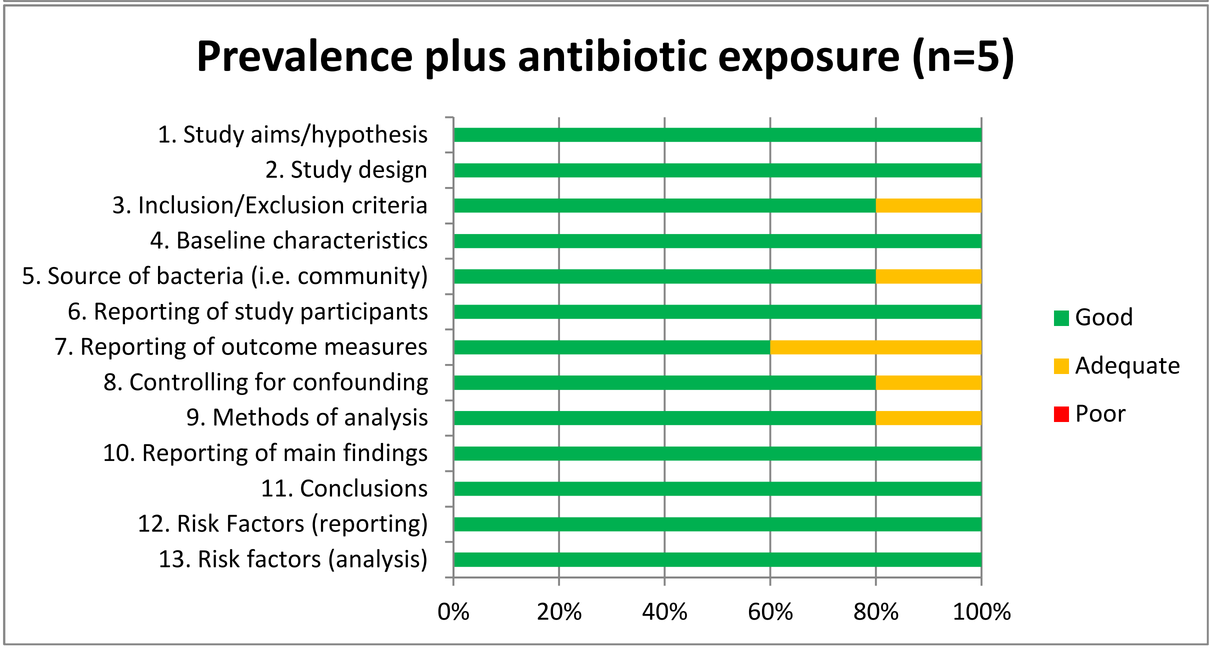

Supplement: Supplementary file 2 — Appendix 2: Data quality charts (by studies reporting prevalence of resistance only and prevalence plus antibiotic exposure) [file brya027820.ww2_default.pdf]
