# Supplementary material for: Global prevalence of antibiotic resistance in paediatric urinary tract infections caused by Escherichia coli and association with routine use of antibiotics in primary care: systematic review and meta-analysis
Source: BMJ. 2016 Mar 15;352:i939. doi: 10.1136/bmj.i939 (PMC4793155; doi:10.1136/bmj.i939)
Supplement: Supplementary file 3 — Appendix 3: Study characteristics [file brya027820.ww3_default.pdf]

Appendix 3: Study characteristics table [posted as supplied by author]

Ref. No.	First author (year)	Country	Design	Recruitment	n	Age range	Sampling method	Antibiotic susceptibilities	Guidelines used to interpret sensitivities	Reported first-line UTI antibiotics?	Previous exposure to antibiotics reported?	Variables adjusted for in antibiotic exposure analysis
**OECD**												
8	Gaspari (2005)	USA	RO	OP	12,731	0-17 yrs	NR	AMP, CZ, CIP, NIT, COT, COA	CLSI	Six most common UTI treatments in USA reported	N	-
20	Edlin (2013)	USA	RO	ED, OP	25,418	0-17 yrs	NR	COT, AMP, COA, NIT, CL, CFX, CZ, CFZ, GEN, CIP, IMI, CFU, PIP-TAZ, AZT	CLSI	COT or cephalosporin	N	-
21	Falagas (2008)	Greece	RO	PC	2460	0-14 yrs	NR	AMP, COA, CC, CP, COT, AMI	CLSI	COT (IDSA guidelines)	N	-
22	Conway (2007)	USA	RO	PC	74,974	0-6 yrs	C, B	-	CLSI	NR	Y	Gender, race, age
23	Fritzsche (2005)	Switzerland	RO	H[a]	354	0-15 yrs	MSU, B, C	AMP, COA, COT, CL, CFU, CFX,	CLSI	NR	N	-
24	Duffy (2013)	UK	RO	PC	1855[b]	0-16 yrs	NR	TRI	NR	TRI	Y	Gender, year urine sample was taken, number of isolates in study period (2004-2009)
25	Yolbas (2013)	Turkey	RO	PC	113	0-15 yrs	MSU, B	AMI, COA, AMP, AMP-SUL, AZT, CZ, CF, CTX, CX, CFZ, CFX, CFU, CIP, ERT, GEN, IMI, LEV, MER, NIT, NOR, PIP, PIP-TAZ, TET, TOB, COT	CLSI	NR	N	-
26	Cullen	Ireland	RO	PC	13,034	0-14 yrs	MSU	AMP, TRI, CIP, COA, CFU,	CLSI	TRI or NIT	N	-

Ref. No.	First author (year)	Country	Design	Recruitment	n	Age range	Sampling method	Antibiotic susceptibilities	Guidelines used to interpret sensitivities	Reported first-line UTI antibiotics?	Previous exposure to antibiotics reported?	Variables adjusted for in antibiotic exposure analysis
	(2013)							GEN, NIT,				
27	Guner (2012)	Turkey	RO	OP	1564	0-17 yrs	MSU, B	AMI, COA, AMP, GEN, IMI, NIT, CZ, CFP, CXM, CFP, CTX, CFX, CFU, CIP, COT	CLSI	AMP or COT or 1st generation cephalosporin	N	-
28	Mantadakis (2011)	Greece	RO	PD	430	0-14 yrs	MSU, C, SA	AMP, COA, TIC, TIC-CLA, CL, CX, CTX, CFZ, IMI, GEN, TOB, NET, AMI, NA, CIP, NOR, OFL, NIT, COT	CLSI	NR	N	-
29	Caracciolo (2011)	Italy	RO	OP	177	0-3 yrs	MSU, C	AMP, COT, COA, CFZ, GEN	CLSI	COT or COA	N	-
30	Paschke (2010)	USA	RO	PD	426	0-6 yrs	MSU, C	AMP, COT, COA	NR	NR	N	-
31	Senel (2010)	Turkey	RO	H[a]	2562	0-16 yrs	MSU, C, SA	AMI, NET, GEN, CIP, CZ, CL, CFU, CFZ, CFX, CF, CFP, AMP, COA, AMP-SUL, COT, PIP, IMI, AZT	CLSI	COT (WHO guidelines)	N	-
32	Catal (2009)	Turkey	RO	PC	204	0-14 yrs	MSU, C, B	AMP, COT, GEN, CFU, AMI, CTX, CIP, IMI, PIP, NIT	CLSI	AMP or aminoglycosides	N	-
33	Prelog (2008)	Austria	RO	H[a]	478	0-15 yrs	MSU, C	AMP, COA, CZ, CXM, CFX, CTX, CFU, CIP, GEN, IMI, NIT, TRI, COT	NR	AMP or COA or CZ or CXM or TRI or COT	N	-
34	Gaspari (2006)	USA	RO	PC	11,341	0-17 yrs	MSU, B, C	AMP, COA, CZ, COT	CLSI	NR	N	-
35	Mehr (2004)	Australia	RO	ED	90	0-6 yrs	MSU, C, SA	AMP, TRI, CL, NIT, CIP, GEN	CLSI	TRI or COT	N	-
36	Burman (2003)	USA	RO	OP	137	0-16 yrs	NR	COT	CLSI	COT	N	-
37	Ladhani (2003)	UK	RO	PC	1774	0-15 yrs	NR	AMP, CD, CFU, COA, GEN, CIP, NIT, TRI	BSAC	NR	N	-
38	McLoughlin (2003)	USA	RO	ED	126	0-17 yrs	MSU, C	COT, AMP, AMP-SUL, CZ, NIT	CLSI	COT	Y	None
39	Ashkenazi	Israel	RO	ED, OP	160	0-14 yrs	MSU, B, C,	SUL, COT, NA, NIT, GEN,	CLSI	COT or AMP	N	-

Ref. No.	First author (year)	Country	Design	Recruitment	n	Age range	Sampling method	Antibiotic susceptibilities	Guidelines used to interpret sensitivities	Reported first-line UTI antibiotics?	Previous exposure to antibiotics reported?	Variables adjusted for in antibiotic exposure analysis
	(1991)						SA	AMP, PIP, CL, CFU, CFZ		or 1st generation cephalosporin		
40	Gruneberg (1976)	UK	RO	OP	37	0-16 yrs	MSU	AMP, STR, TET, CHL, SUL	NR	NR	N	-
41	Bryce (2010)	UK	RO	PC	1746	0-10 yrs	MSU	AMP, AMI, CFZ, CLX, COA, CTX, GEN, NA, NIT, NOR, TOB, TRI, CIP	CLSI	TRI or NIT	N	-
42	McGregor (2014)	USA	RO	OP	3681	0-15 yrs	NR	AMP, COA, CZ, CL, CIP, GEN, NIT, TET, TOB, COT	NR	COT (AAP guidelines)	N	-
43	Swerkersson (2014)	Sweden	RO	ED	928	0-2 yrs	MSU, C, B, SA	TRI, NIT, CD	CLSI	TRI	N	-
44	Anatoliotaki (2007)	Greece	PO	H[a]	208	0-14 yrs	MSU, C, SA	AMP, COA, CC, CFU, COT, NIT, GEN	CLSI	COA or COT	N	-
45	Gallegos (2013)	Chile	PO	ED	100	0-5 yrs	C	AMP-SUL, CL, CFZ, CTX, GEN, CIP, COT, NIT	NR	Cephalosporin	N	-
46	Ismaili (2011)	Belgium	PO	ED	189	0-17 yrs	MSU, C, SA	AMP, COA, COT, NIT, CFU, CFZ, CTX, CFX, CIP, GEN, AMI, TEM	NR	AMP or COT	N	-
47	Ipek (2011)	Turkey	PO	OP	103	0-14 yrs	MSU, C	AMP, COA, CZ, CFU, CXM, CF, AZT, IMI, GEN, CIP, NIT, COT	CLSI	NIT or CXM	N	-
48	Yildirim (2010)	Turkey	PO	OP	109	0-17 yrs	NR	COT, AMP, COA, CC, CIP, CFX, CFZ, IMI, PIP, AMI, GEN	CLSI	COT	N	-
49	Borsari (2008)	Switzerland	PO	OP	100	0-17 yrs	MSU, C	AMP, CPD, COA, COT, NIT	CLSI	COT or COA or cephalosporin	N	-
50	Allen (1999)	USA	CC	PD	967	0-6 yrs	NR	AMP, COT, GEN, CZ, CTX, NIT, NOR, TIC	CLSI	COT	Y	Gender, age, genitourinary tract abnormalities, previous hospital admissions, genitourinary

Ref. No.	First author (year)	Country	Design	Recruitment	n	Age range	Sampling method	Antibiotic susceptibilities	Guidelines used to interpret sensitivities	Reported first-line UTI antibiotics?	Previous exposure to antibiotics reported?	Variables adjusted for in antibiotic exposure analysis
												reflux, malignant disorder.
51	Topaloglu (2010)	Turkey	CC	OP	4105	0-16 yrs	MSU, B, C, SA	-	CLSI	NR	Y	Renal abnormalities, history of systemic diseases, previous hospitalisation
**Non-OECD**												
52	Alshara (2011)	Jordan	RO	H[a], OP	435	0-15 yrs	NA	AMI, COA, AMP, CFZ, CLX, CXM, CIP, CL, COT, CFX, CTX, GEN, NA, NOR	NR	COT or NIT or cephalosporin	N	-
53	Al-Saif (2012)	Saudi-Arabia	RO	PC	175	0-14 yrs	MSU, B, C	AMP, CFU, NIT, COT, CTX, CFZ, CFX, GEN, AMI, TAZ, IMI	CLSI	AMP or COT	N	-
54	Valavi (2013)	Iran	RO	H[a]	242	0-15 yrs	MSU, C, SA	COT, AMI, GEN, NA, CFX, CTX, CXM, NIT	CLSI	COT	N	-
55	Muoneke (2012)	Nigeria	RO	OP	15	0-5 yrs	NR	GEN, CFX, CIP, CFZ, CFU, CTX	NR	CIP	N	-
56	Farshad (2011)	Iran	RO	OP	90	0-14 yrs	NR	AMP, COT, TET, CHL, NA, CXM, CFU, GEN, CFZ, CIP, NOR	CLSI	NR	N	-
57	Al-Mardeni (2009)	Jordan	RO	OP	429	0-14 yrs	NR	AMP, COT, NA, CLX, NIT, CFX, GEN,	CLSI	COT or CLX (AAP guidelines)	N	-
58	Guidoni (2008)	Brazil	RO	PD	206	0-15 yrs	MSU, C	NIT, NA, NOR, CIP, AMI, AMP, COT	CLSI	COT or AMP (WHO guidelines)	N	-
59	Rai (2008)	Nepal	RO	OP	502	0-15 yrs	MSU	NIT, NA, AMI, OFL, CIP, COT, CFX, CTX, CLX, NOR, CHL, GEN	NR	NR	N	-
60	Goldraich (2002)	Brazil	RO	ED	225	1-12 yrs	SA	NIT, NA, CLX, COT	CLSI	NR	N	-
61	Sharef	Oman	RO	PC	120	0-14 yrs	MSU, C	AMP, COA, AMI, CIP, CTX,	NR	COT (AAP	N	-

Ref. No.	First author (year)	Country	Design	Recruitment	n	Age range	Sampling method	Antibiotic susceptibilities	Guidelines used to interpret sensitivities	Reported first-line UTI antibiotics?	Previous exposure to antibiotics reported?	Variables adjusted for in antibiotic exposure analysis
	(2015)							CFX, CFU, NIT, GEN, MER, COT, TAZ		guidelines)		
62	Sharan (2013)	India	PO	NR	49	0-5 yrs	SA	AMP, COA, CLX, CIP, NOR, OFL, AZI, LEV, NIT, AMI, CFZ, CFU, GEN, IMI, MER, CTX, CFX, PIP-TAZ	NR	NR	N	-
63	Mandal (2012)	India	PO	OP	406	0-17 yrs	MSU, C, SA	CIP	CLSI	COT (IDSA guidelines)	N	-
64	Sharma (2011)	Nepal	PO	OP	27	0-15 yrs	MSU	CTX, OFL, NOR, NIT, NA, CIP, AMI, AMP, COT	CLSI	Quinolones	N	-
65	Wu (2012)	Taiwan	PO	H[a]	136	0-4 mth	C, B, SA	AMP, CZ, GEN, COT, CFX	CLSI	NR	N	-
66	Farshad (2012)	Iran	PO	H[a]	96	0-14 yrs	NR	AMP, COT, TET, NA, CXM, GEN, CIP, NIT, AMI, IMI	CLSI	NR	N	-
67	Pourakbari (2012)	Iran	PO	PC	50	0-12 yrs	MSU, C, SA	COT, GEN, CL, CFU, CXM, AMI, NIT, MER, CFX	CLSI	COT	N	-
68	Farshad (2010)	Iran	PO	OP	96	0-14 yrs	NR	AMP, NA, CXM, GEN, CIP, NIT, AMI, IMI	CLSI	NR	N	-
69	Adjei (2004)	Ghana	PO	H[a]	15	0-1 yr	SA	AMP, COA, COT, NIT, CFU	CLSI	COT or AMP	N	-
70	Al-Mugeiren (1996)	Saudi-Arabia	PO	PC	596	0-12 yrs	MSU	AMP, SUL, TRI, NIT, GEN, COA, CFU, CFZ	NR	NR	N	-
71	Helin (1986)	Kuwait	PO	OP	67	0-12 yrs	NR	AMP	CLSI	AMP	N	-
72	Narchi (2008)	United Arab Emirates	PO	H[a]	208	0-12 yrs	MSU, C, SA	AMP, COA, CLX, CFU, GEN, COT, NIT, NA	CLSI	Cephalosporin	N	-
73	Brown (2003)	Nigeria	CC	OP	17	1-15 yrs	MSU	AMP, GEN, CFX, CFZ, CFU, NIT, NA, COA, COT	NR	COT or AMP	N	-
74	Fredrick (2013)	Tanzania	CS	H[a]	25	0-5 yrs	C, SA	COT, AMP, COA, CFX, GEN, AMI	CLSI	AMP or COT (WHO guidelines)	N	-
75	Afsharpaiman (2012)	Iran	CS	H[a]	81	0-2 yrs	MSU, SA	CTX, CLX, CXM, CFZ, AMI, GEN, CHL, NA, NIT	CLSI	NR	N	-
76	Sedighi	Iran	CS	H[a]	100	0-5 yrs	MSU, C,	COT, CXM, NA, NIT, CFX,	CLSI	COT	N	-

Ref. No.	First author (year)	Country	Design	Recruitment	n	Age range	Sampling method	Antibiotic susceptibilities	Guidelines used to interpret sensitivities	Reported first-line UTI antibiotics?	Previous exposure to antibiotics reported?	Variables adjusted for in antibiotic exposure analysis
	(2014)						SA	AMI				

Where - = not applicable

Design: RO = retrospective observational; PO = prospective observational; CC = case-control; CS = cross-sectional.

Recruitment: OP = outpatient department; PC = primary care centre/community practice; ED = emergency department; H = hospital admission; LR = laboratory records; PD = paediatric physician visit.

Sampling method: MSU = midstream urine/clean-catch; B = urine bags; C = catheter; SA = suprapubic aspiration; NR = not reported

Antibiotic susceptibilities: AMI = amikacin; AMP = ampicillin; AMP-SUL = ampicillin-sulbactam; AZI = azithromycin; AZT = aztreonam; CC = cefaclor; CD = cedadroxil; CF = cefepime; CFP = cefoperazone; CFU = cefuroxime; CFX = ceftriaxone; CFZ = ceftazidime; CHL = chloramphenicol; CIP = ciprofloxacin; CL = cefalothin; CLX = cefalexin; COA = co-amoxiclav; COT = co-trimoxazole; CP = cefprozil; CTX = cefotaxime; CX = cefoxitin; CXM = cefixime; CZ = cefazolin; GEN = gentamicin; IMI = imipenem; LEV = levofloxacin; MER = meropenem; NA = naladixic acid; NET = netilmicin; NIT = nitrofurantoin; NOR = norfloxacin; OFL = ofloxacin; PIP = piperacillin; PIP-TAZ = piperacillin-tazobactam; STR = streptomycin; SUL = sulfamethoxazole; TAZ = tazobactam; TEM = temocillin; TET = tetracycline; TIC = ticarcillin; TRI = trimethoprim

Guidelines: CLSI = Clinical & Laboratory Standards Institute; BSAC = British Society for Antimicrobial Chemotherapy; NR = not reported

First-line antibiotics: based on reporting of first-line antibiotics used for paediatric UTI in the paper. Guidelines referred to include Infectious Disease Society of America (IDSA), World Health Organisation (WHO) and the American Academy of Pediatrics (AAP).

[a] For all studies recruiting hospital inpatients: reference 33, 44 and 66 were recruited and sampled on admission, sample confirmed as taken within 48hrs; reference 23, 31, 52, 54, 65, 69, 72, 74, 75 and 76 recruited confirmed CA-UTI cases on admission.

[b] For all studies except reference 24, only one urine sample per child was analysed. Reference 24 included multiple urine samples per child recruited to the study.
