# Supplementary material for: Global prevalence of antibiotic resistance in paediatric urinary tract infections caused by Escherichia coli and association with routine use of antibiotics in primary care: systematic review and meta-analysis
Source: BMJ. 2016 Mar 15;352:i939. doi: 10.1136/bmj.i939 (PMC4793155; doi:10.1136/bmj.i939)
Supplement: Supplementary file 4 — Appendix 4: Supplementary forest plots [file brya027820.ww4_default.pdf]

Appendix 4: Supplementary forest plots [posted as supplied by author]

Ampicillin resistance in *E. coli* urinary isolates from children, by OECD status

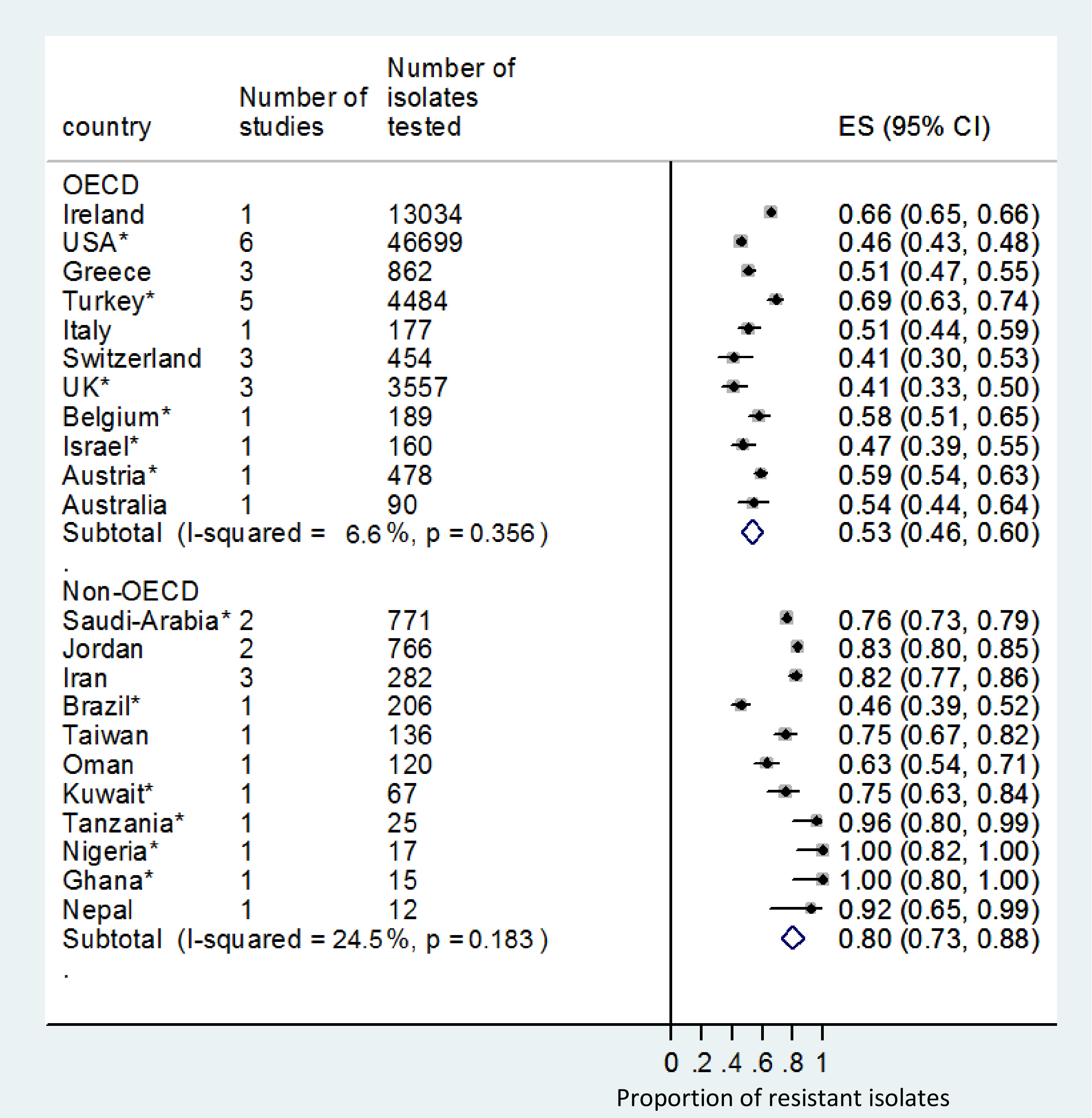

country	Number of studies	Number of isolates tested	ES (95% CI)
OECD			
Ireland	1	13034	0.66 (0.65, 0.66)
USA*	6	46699	0.46 (0.43, 0.48)
Greece	3	862	0.51 (0.47, 0.55)
Turkey*	5	4484	0.69 (0.63, 0.74)
Italy	1	177	0.51 (0.44, 0.59)
Switzerland	3	454	0.41 (0.30, 0.53)
UK*	3	3557	0.41 (0.33, 0.50)
Belgium*	1	189	0.58 (0.51, 0.65)
Israel*	1	160	0.47 (0.39, 0.55)
Austria*	1	478	0.59 (0.54, 0.63)
Australia	1	90	0.54 (0.44, 0.64)
Subtotal (I-squared = 6.6%, p = 0.356)			0.53 (0.46, 0.60)
.			
Non-OECD			
Saudi-Arabia*	2	771	0.76 (0.73, 0.79)
Jordan	2	766	0.83 (0.80, 0.85)
Iran	3	282	0.82 (0.77, 0.86)
Brazil*	1	206	0.46 (0.39, 0.52)
Taiwan	1	136	0.75 (0.67, 0.82)
Oman	1	120	0.63 (0.54, 0.71)
Kuwait*	1	67	0.75 (0.63, 0.84)
Tanzania*	1	25	0.96 (0.80, 0.99)
Nigeria*	1	17	1.00 (0.82, 1.00)
Ghana*	1	15	1.00 (0.80, 1.00)
Nepal	1	12	0.92 (0.65, 0.99)
Subtotal (I-squared = 24.5%, p = 0.183)			0.80 (0.73, 0.88)
.			

ES = Effect size
Ordered by increasing standard error
*Indicates antibiotic is first-line treatment choice

Co-amoxiclav resistance in *E. coli* urinary isolates from children, by OECD status

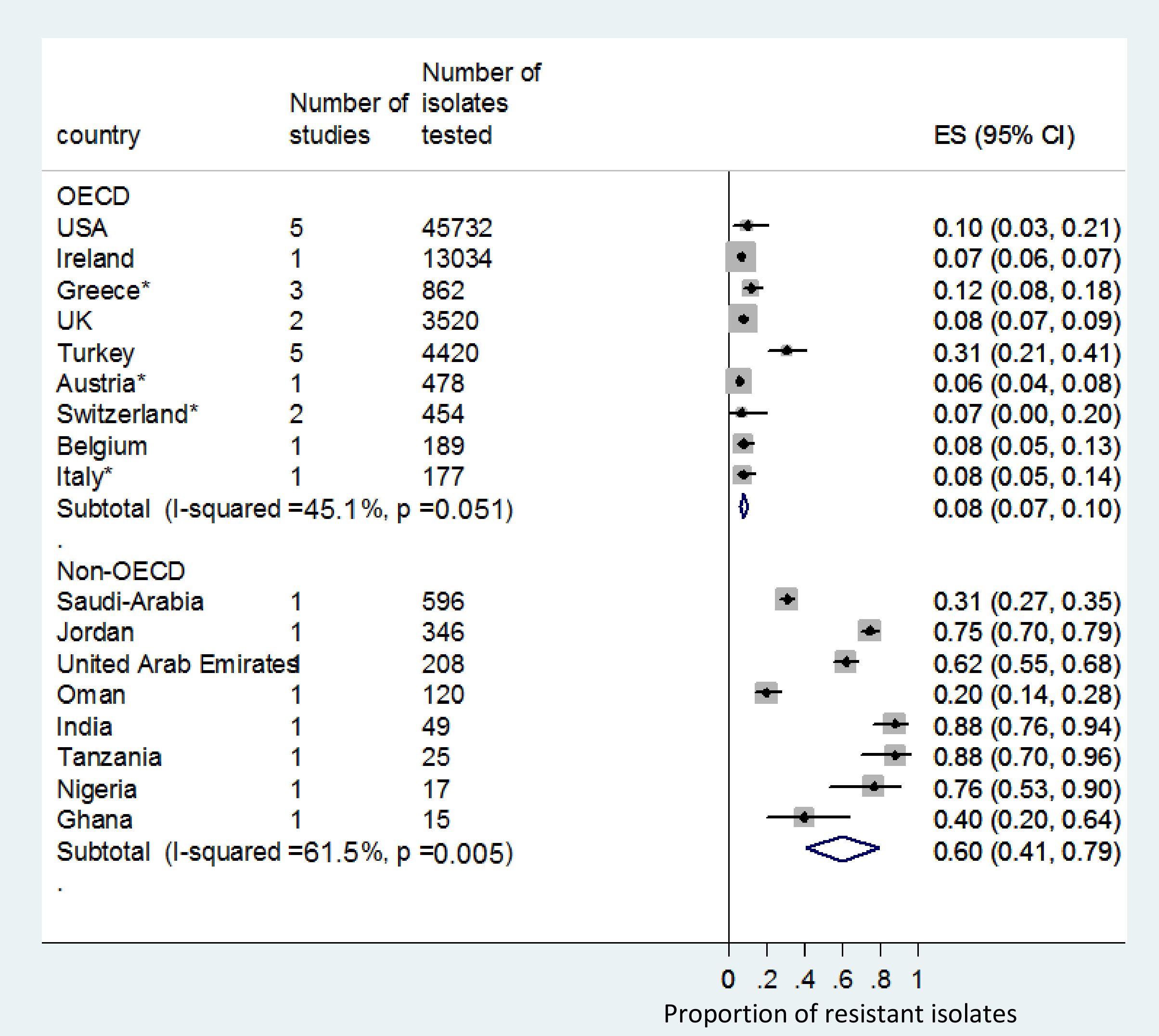

ES = Effect size

Ordered by increasing standard error

*Indicates antibiotic is first-line treatment choice

Co-trimoxazole resistance in *E. coli* urinary isolates from children, by OECD status

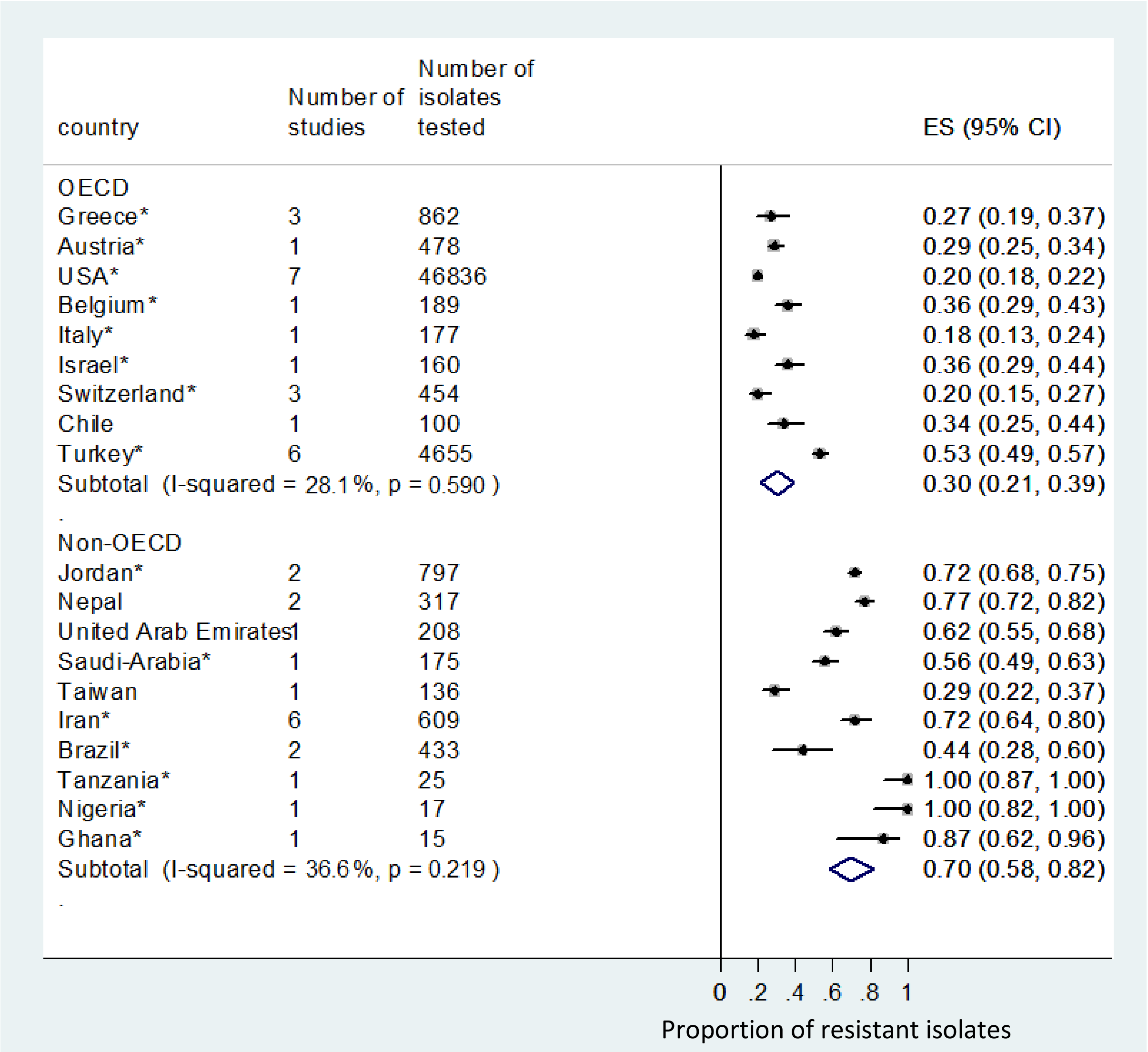

ES = Effect size

Ordered by increasing standard error

*Indicates antibiotic is first-line treatment choice

Trimethoprim resistance in *E. coli* urinary isolates from children, by OECD status

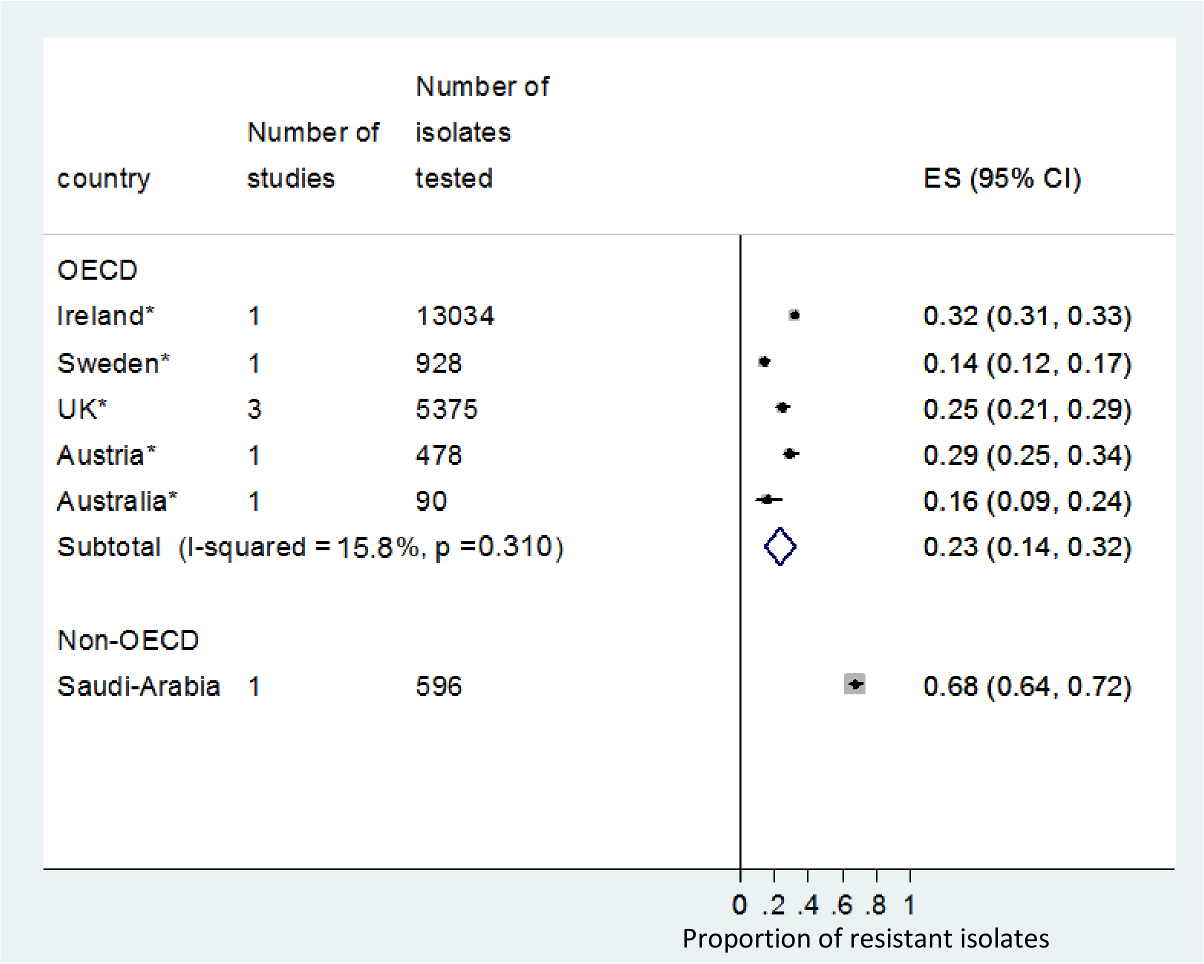

ES = Effect size
Ordered by increasing standard error
*Indicates antibiotic is first-line treatment choice

Nitrofurantoin resistance in *E. coli* urinary isolates from children, by OECD status

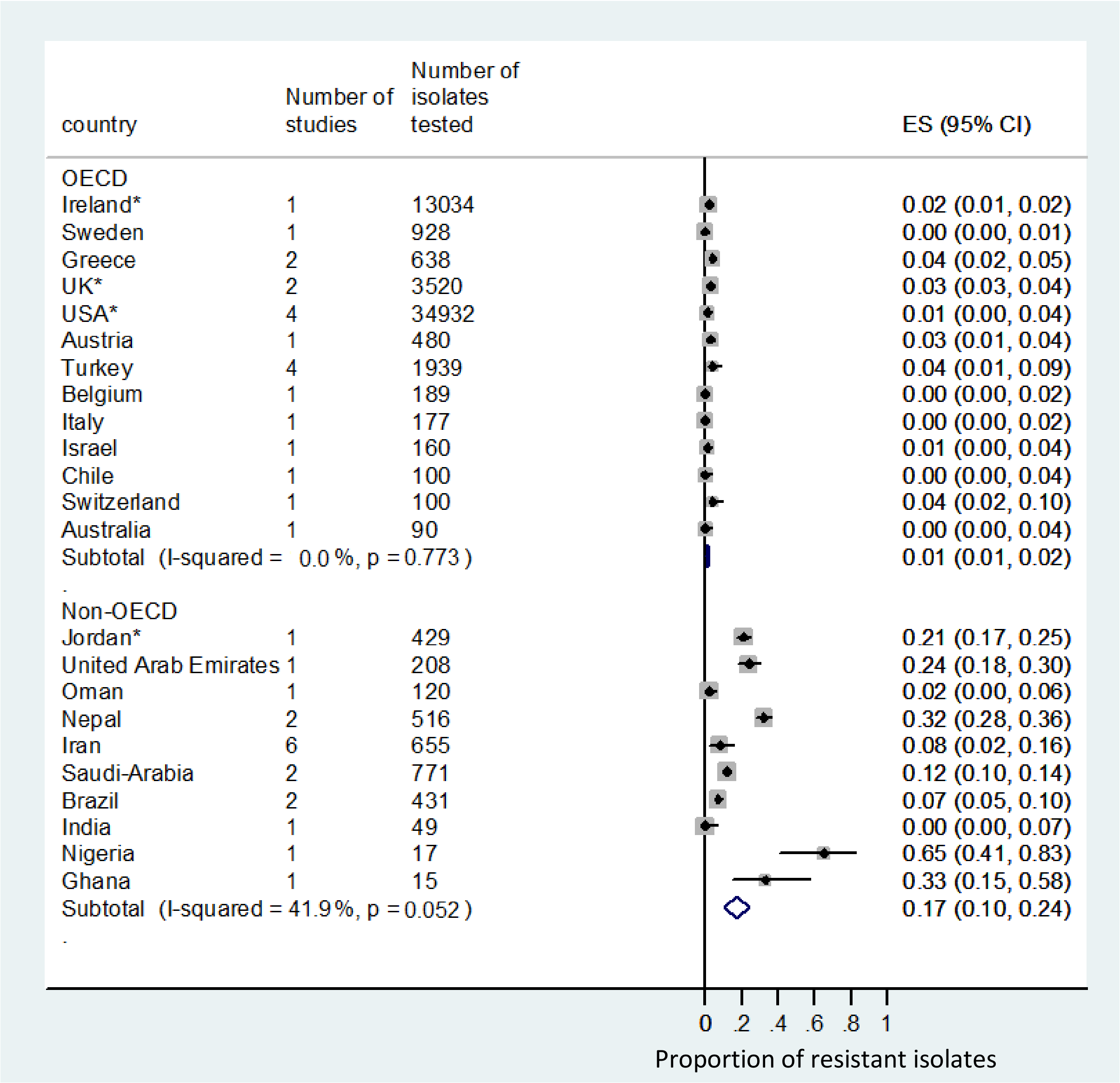

ES = Effect size

Ordered by increasing standard error

*Indicates antibiotic is first-line treatment choice

Ciprofloxacin resistance in *E. coli* urinary isolates from children, by OECD status

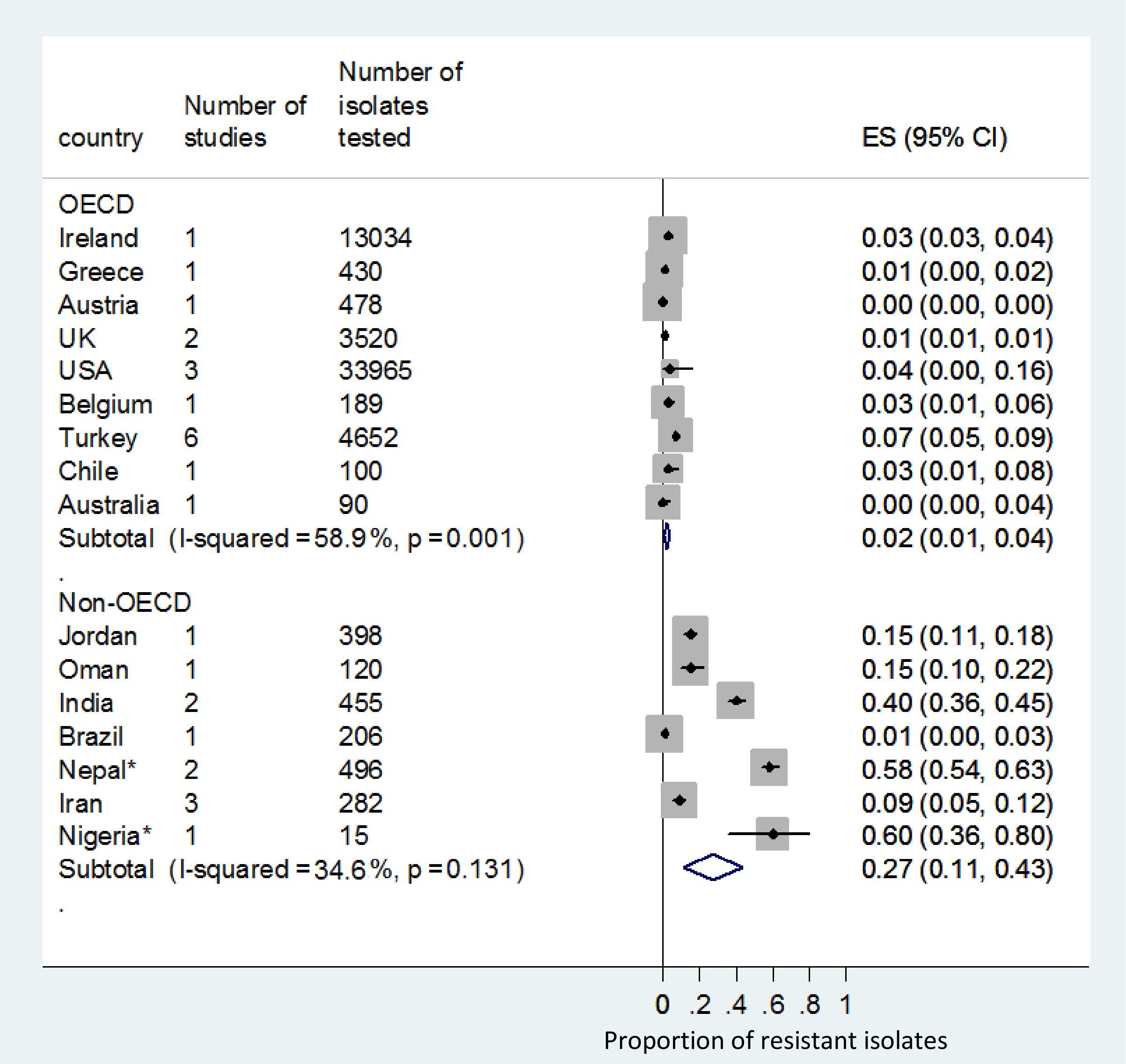

ES = Effect size
Ordered by increasing standard error
*Indicates antibiotic is first-line treatment choice

Ceftazidime resistance in *E. coli* urinary isolates from children, by OECD status

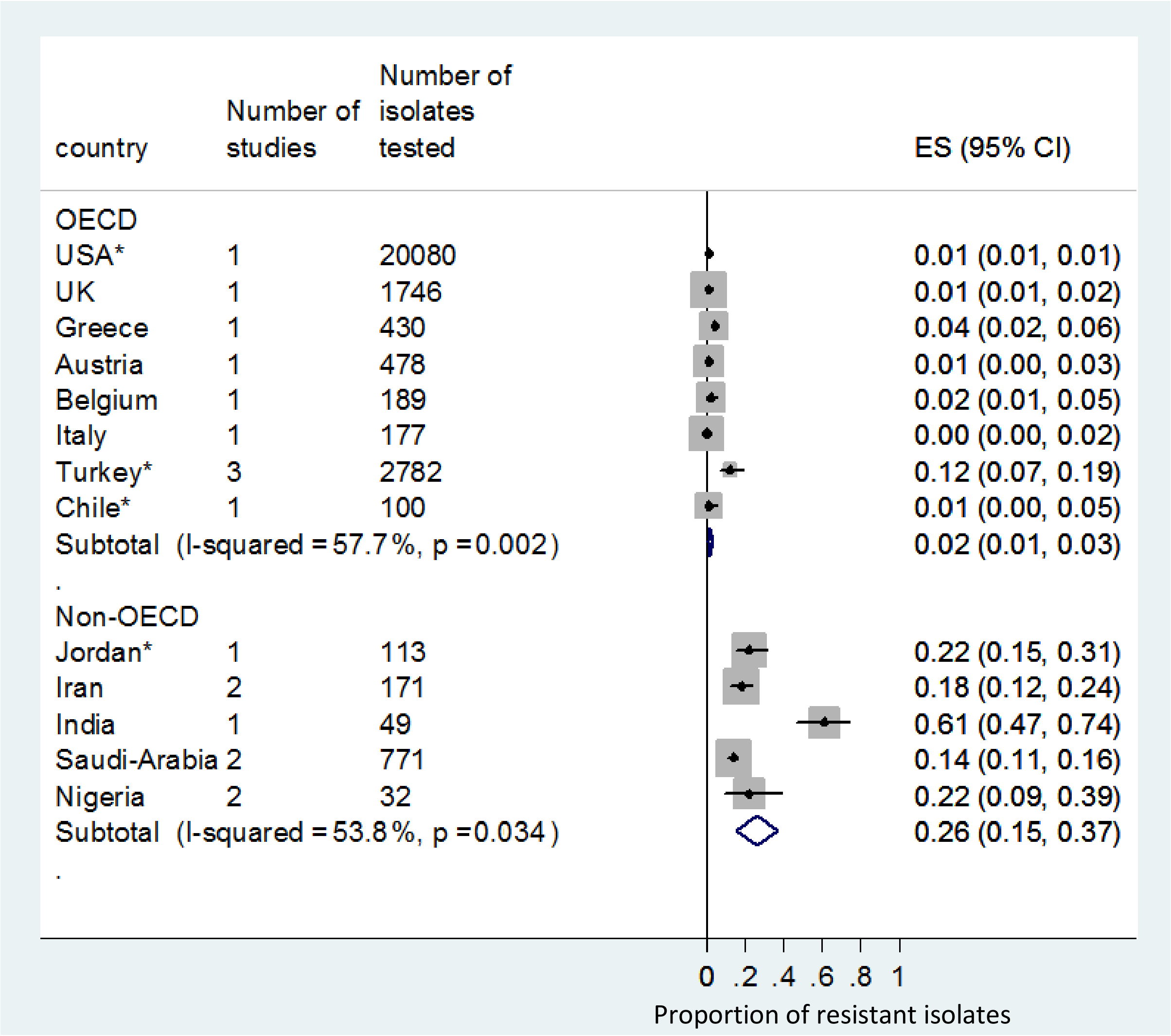

ES = Effect size

Ordered by increasing standard error

*Indicates antibiotic is first-line treatment choice
