# Supplementary material for: Global prevalence of antibiotic resistance in paediatric urinary tract infections caused by Escherichia coli and association with routine use of antibiotics in primary care: systematic review and meta-analysis
Source: BMJ. 2016 Mar 15;352:i939. doi: 10.1136/bmj.i939 (PMC4793155; doi:10.1136/bmj.i939)
Supplement: Supplementary file 5 — Appendix 5: Prevalence of resistance by country level reported first line antibiotic treatment for urinary tract infection in OECD and non-OECD countries [file brya027820.ww5_default.pdf]

Appendix 5: Prevalence of resistance by country-level reported first-line antibiotic treatment for UTI in OECD (a) and non-OECD (b) countries

(a) OECD

Antibiotics	Reported[a] first-line UTI treatment				First-line UTI treatment not specified			
	Pooled prevalence (%)	Number of isolates tested	Number of reporting studies	I^2 (%)	Pooled prevalence (%)	Number of isolates tested	Number of reporting studies	I^2 (%)
**Ampicillin**	54.0 (44.5-63.4)	55,567	17 (6 countries)	69.6	52.7 (40.9-64.4)	14,617	8 (5 countries)	3.0
**Co-amoxiclav**	9.4 (5.9-12.9)	1971	7 (4 countries)	1.1	10.8 (7.7-14.0)	66,895	14 (5 countries)	0.0
**Co-trimoxazole**	31.5 (20.3-42.7)	53,634	23 (8 countries)	10.6	34.0 (24.9-44.1)	277	1 (1 country)	-
**Trimethoprim**	23.6 (17.9-30.3)	18,977	7 (5 countries)	11.5	-	0	0	-
**Nitrofurantoin**	2.4 (1.7-3.1)	51,486	7 (3 countries)	0.0	1.0 (0.2-1.8)	4801	14 (10 countries)	20.6
**Ciprofloxacin**	-	0	0	-	3.1 (0.8-5.4)	52,209	17 (9 countries)	0.0
**Ceftazidime[b]**	3.1 (0.1-11.3)	22,962	5 (3 countries)	0.0	1.2 (0.4-2.0)	3020	5 (5 countries)	47.7

[a] Reported first-line treatment defined as either recommended as part of national/international guidelines or the antibiotic that would be given by a pharmacist when presenting with UTI symptoms.

[b] Ceftazidime reported as a marker for cephalosporin resistance, where any cephalosporin was reported as a first-line treatment.

(b) Non-OECD

Antibiotics	Reported[a] first-line UTI treatment				First-line UTI treatment not specified			
	Pooled prevalence (%)	Number of isolates tested	Number of reporting studies	I^2 (%)	Pooled prevalence (%)	Number of isolates tested	Number of reporting studies	I^2 (%)
**Ampicillin**	84.2 (67.5-96.0)	1101	7 (6 countries)	0.0	77.9 (70.6-85.2)	1316	8 (5 countries)	39.6
**Co-amoxiclav**	-	0	0	-	60.3 (40.9-79.0)	1256	8 (8 countries)	69.7
**Co-trimoxazole**	76.2 (64.1-87.2)	2071	14 (7 countries)	7.3	55.6 (26.6-84.7)	661	4 (3 countries)	0.0
**Trimethoprim**	-	0	0	-	67.0 (64.4-72.4)	596	1 (1 country)	-
**Nitrofurantoin**	21.2 (17.0-24.8)	429	1 (1 country)	-	13.7 (7.6-19.7)	2782	17 (9 countries)	27.0
**Ciprofloxacin**	58.1 (51.5-64.7)	511	3 (2 countries)	0.0	15.8 (4.7-26.8)	1461	8 (5 countries)	26.3
**Ceftazidime[b]**	22.3 (15.4-31.2)	113	1 (1 country)	-	24.2 (13.5-35.0)	1023	7 (4 countries)	63.1

[a] Reported first-line treatment defined as either recommended as part of national/international guidelines or the antibiotic that would be given by a pharmacist when presenting with UTI symptoms.

[b] Ceftazidime reported as a marker for cephalosporin resistance, where any cephalosporin was reported as a first-line treatment.
